# The Genomic Health of Human Pluripotent Stem Cells: Genomic Instability and the Consequences on Nuclear Organization

**DOI:** 10.3389/fgene.2018.00623

**Published:** 2019-01-21

**Authors:** Marianne P. Henry, J. Ross Hawkins, Jennifer Boyle, Joanna M. Bridger

**Affiliations:** ^1^Advanced Therapies Division, National Institute for Biological Standards and Control, Potters Bar, United Kingdom; ^2^Laboratory of Nuclear and Genomic Health, Division of Biosciences, Department of Life Sciences, College of Health and Life Sciences, Brunel University London, London, United Kingdom

**Keywords:** aneuploidy, genome, stem cell, chromosome, nucleus (positioning)

## Abstract

Human pluripotent stem cells (hPSCs) are increasingly used for cell-based regenerative therapies worldwide, with embryonic and induced pluripotent stem cells as potential treatments for debilitating and chronic conditions, such as age-related macular degeneration, Parkinson's disease, spinal cord injuries, and type 1 diabetes. However, with the level of genomic anomalies stem cells generate in culture, their safety may be in question. Specifically, hPSCs frequently acquire chromosomal abnormalities, often with gains or losses of whole chromosomes. This review discusses how important it is to efficiently and sensitively detect hPSC aneuploidies, to understand how these aneuploidies arise, consider the consequences for the cell, and indeed the individual to whom aneuploid cells may be administered.

## Introduction

Stem cells are unspecialized cells that can give rise to a ranged of different cell types through self-renewal. Adult (mesenchymal) stem cells (MSCs) can be found throughout the body in various niches, such as the small intestine, colon or bone marrow (Barker et al., 2007; Hérault et al., 2017). Embryonic stem cells (ESCs) on the other hand are derived from the inner cell mass of an early preimplantation embryo or blastocyst and can differentiate to form all three germ cell layers. Such cells are known as pluripotent cells, since they give rise to every cell type of the body, excluding the extra-embryonic membrane and placental tissue. With such immense therapeutic potential, stem cells could be used for tissue repair and potentially replacement of whole organs through tissue engineering, circumventing the problem of a current lack of organ donors (Badylak et al., 2011). Due to their pluripotent properties, the treatment of many diseases such as age-related macular degeneration (Song et al., 2015), spinal cord injuries (Deshpande et al., 2006), type 1 diabetes (Farooq et al., 2018), and Parkinson's disease (Bjorklund et al., 2002; Takagi et al., 2005; Grealish et al., 2014; Barker et al., 2016) may soon become a reality.

Induced pluripotent stem cells (iPSCs) are pluripotent cells generated by the reprogramming of differentiated cells and can likewise give rise to a range of different cell types. iPSCs may be considered as the ideal therapeutic resource since an autologous stem cell transplant negates the need for human leukocyte antigen (HLA) matching and any immunosuppression required with allogenic transplants, as well as providing an endless supply of personalized therapeutic product if required. It has been estimated that a relatively small number of iPSC lines need be generated to meet a demand that covers most of the world's population via the generation of HLA matched banks, making it both cost-effective and simpler for thorough characterization from a regulatory perspective (Taylor et al., 2012; Turner et al., 2013; Solomon et al., 2015). iPSCs are created from differentiated cells and can be reprogrammed to become pluripotent mainly through three genes: *OCT4, SOX2*, and *NANOG*, which induce and maintain the upregulation of pluripotency genes whilst repressing lineage-associated genes.

**Figure 1:**
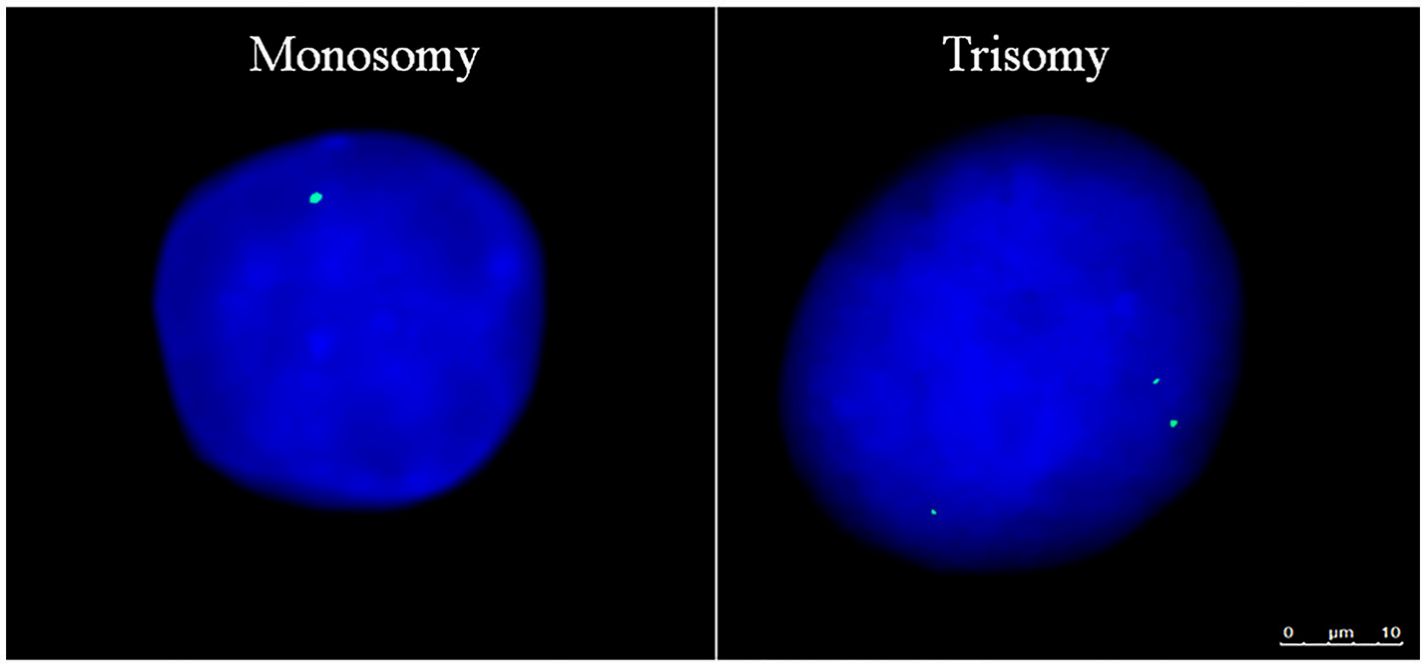
Aneuploid Gene Loci within Human Embryonic Stem Cells. Aneuploid pluripotent stem cell nuclei subjected to fluorescence *in-situ* hybridization displaying *AMELX* gene loci in green and nuclear DNA stained with DAPI in blue. Scale bar is 10 μm.

Both ESCs and iPSCs are noted for their accumulation of chromosomal aneuploidies, especially after prolonged *in vitro* culturing (Amps et al., 2011). Similarly, cells of the blastocyst also exhibit a high rate of mitotic aneuploidy (Taylor et al., 2014) and thus it is possible that the chromosomes of pluripotent cells are inherently unstable. Interestingly, in the blastocyst, more chromosome losses than gains are observed (Chung et al., 2013; Yao et al., 2016), in contrast to hESCs having more gains, which may lead to these affected hESCs having a greater selective advantage in cell culture (Amps et al., 2011). Typically hESC chromosome aneuploidies include chromosomes 1, 12, 17, 20, and X (Draper et al., 2004; Maitra et al., 2005; Baker et al., 2007) (Figure 1). This is in contrast to live births, where the most common aneuploidies are for chromosomes containing fewer genes i.e., autosomes 13, 18, and 21 (Caine et al., 2005) along with the sex chromosomes (Munné et al., 1998), and spontaneous abortions, where common aneuploidies include chromosomes 4, 7, 13, 15, 16, 21, and 22 (Fritz et al., 2001) (Table 1). Seemingly the aneuploidies accumulating in the hPSC culture are incompatible with life and are strikingly similar to the aneuploidies found in human embryonal carcinoma cells (hECCs), with respect to the types of karyotypic changes observed (Summersgill et al., 2001; Reuter, 2005; Harrison et al., 2007) and in their gene expression profiles (Sperger et al., 2003), suggesting a tumorigenic potential. Furthermore, stem cells with these recurrent gains or losses display a growth advantage in culture (Amps et al., 2011; Avery et al., 2013; Peterson and Loring, 2014), signifying that these chromosomes contain critical genes needed for cell growth, pluripotency and possibly tumorigenesis. This poses a serious threat to the therapeutic use of hPSCs, as the effects of using genomically abnormal or unstable stem cells in patients is unknown (Brimble et al., 2004; Draper et al., 2004; Peterson and Loring, 2014). Those chromosomal rearrangements common to hESCs and hECCs are candidates as drivers of tumorigenesis. Gene sequence and copy-number mutations affecting known oncogenes may also drive tumorigenesis. Screening oncogenes for mutations in hESCs might therefore become a necessity in providing a risk analysis of hESC lines prior to use in cell therapies. Indeed, in a study of 140 hESC lines, 5 were found to contain mutations in the oncogene *TP53* (Merkle et al., 2017), highlighting the risk of employing hPSCs for cellular therapies.

**Table 1:** Chromosomal abnormalities in specific cell types or in live births and spontaneous abortions.

**Cell type**	**Chromosomal abnormalities**
Embryonic stem cells	1, 12, 17, 20, X
Induced pluripotent stem cells	1, 9, 12, 20, X
Human embryonal carcinoma cells	1, 12, 17, 20, X
Live births	13, 18, 21, X, Y
Spontaneous abortions	4, 7, 13, 15, 16, 21, 22

*Specific chromosome gains and/or losses that occur most commonly in the different cell types, and in live births and spontaneous abortions*.

What effect(s) the hPSC aneuploidies may have, if cells containing them are administered to patients, needs to be addressed. An issue that is particularly important to address is the risk of transplanting hPSCs into individuals without being able to control their self-renewal capacity (Kanemura et al., 2014). The possibility of a malignant transformation of the cells followed by unregulated proliferation could limit stem cells use for future therapies (Blum and Benvenisty, 2008; Herberts et al., 2011; Ben-David et al., 2014). Worryingly, it has already been demonstrated that the transplantation of aneuploid cultured murine MSCs leads to malignant transformation *in vivo* (Miura et al., 2006). This could lead to devastating consequences if patients were recipients of genomically unstable hPSCs. Tumor development from non-host origin has been reported after the injection of karyotypically normal neural stem cells into an Ataxia Telangiectasia patient (Amariglio et al., 2009). Whilst many details of the procedure were not disclosed, it is thought that sufficient genomic characterization of the donor cells was not performed prior to transplantation (Baker, 2009). This case, along with the supporting studies presenting mosaicism (Amps et al., 2011; Merkle et al., 2017) and recurrent chromosomal abnormalities (Brimble et al., 2004; Draper et al., 2004; Baker et al., 2007; Amps et al., 2011) giving rise to growth advantage in culture, highlights the importance of vigorous characterization of the hPSCs before transplantation if such cells were to be used regularly in therapies, and also the need for the development of novel analytics for such characterization.

Additionally, it has been reported that somatic cells with pre-existing chromosomal mutations limited the reprogramming of the cells to iPSCs (Yang C. et al., 2008). However, recent *in vitro* studies, generating hESCs with trisomies of either chromosomes 6, 8, 11, 12, or 15, demonstrate that proliferation may not be the issue, but the ability of stem cells containing aneuploidies to be able to differentiate efficiently and in a timely fashion is (Zhang et al., 2016). These experimentally induced aneuploidies also gave rise to global changes in gene expression profiles, evident in the differentiated somatic cells whereby gene expression alterations were found throughout the genome (Dürrbaum and Storchová, 2016). These technical issues once again demonstrate the inefficiency and potential malignancy of using aneuploid hPSCs in therapies.

It is concerning that aneuploid hPSCs may have a growth advantage *in vivo*, due to the selection of specific gene gains or losses, driving the concomitant gain or loss of part or whole chromosomes e.g., the gain of chromosome 20 in hPSCs driven by the *BCL2L1* gene (Enver et al., 2005; Baker et al., 2007). This gene is associated with anti-apoptotic properties (Boise et al., 1993; Amps et al., 2011; Avery et al., 2013; Na et al., 2014) and is a hallmark of cancer (Herszfeld et al., 2006; Yang S. et al., 2008; Avery et al., 2013). Knock-down of *BCL2L1* diminished the growth advantage effect and thus, this gene is likely to be the driver of chromosome 20 accumulation in hESC cultures (Avery et al., 2013). Following the event that creates aneuploid cells, selection is then required to increase the proportion of aneuploid cells relative to the normal diploid cell population. There are several points during hESC culture at which selection could operate, but evidence points to the mechanism used for disaggregating cells for passaging. For example, aneuploidies were gained when employing enzymatic and non-enzymatic methods of cell dissociation, rather than manual colony cutting in hESC cultures (Mitalipova et al., 2005). Furthermore, aneuploid cells showed an increase in the expression of pluripotency genes and early differentiation genes, implying that the cell disaggregation method may induce widespread changes in the phenotype of the cell culture. Candidate genes suggested to infer a growth advantage include the pluripotency–related genes *NANOG, DPPA3*, and *GDF3*, oncogene *KRAS*, and cell cycle regulator *CCND2* on chromosome 12, and *BIRC5 (SURVIVIN)* on chromosome 17 (Na et al., 2014). It is also possible that mutation-bearing cells with no selective advance in culture may become present at significant levels to chance-effects in the bottleneck created by colony-cutting and poor cell survival rates upon passage. However, with the limitations of current analytics, it is difficult to discern the precise levels of aneuploidies appearing in culture.

In this article, we will review the mechanisms by which aneuploidies may arise in hPSCs, and the potential impact on genome organization and stability, concluding with an analysis on the current tools available to measure genomic aberrations toward ensuring safe therapeutic application.

## How Aneuploidies Arise

In order to maintain genomic integrity, it is essential that with each cell division the distribution of chromosomes in each daughter cell is matched. Unfortunately, how exactly aneuploidies arise in human pluripotent stem cells is not yet entirely known. We discuss here a number of mechanisms that could lead to the formation of aneuploidies and discuss the genomic abnormalities that may contribute to aneuploidy status.

### Mitotic Segregation Defects

Telomeres are repetitive nucleotide sequences found at the end of chromosomes to prevent chromosome end-to-end fusions, which can result in chromosome instability. Normally, telomeres shorten as a result of each cell division, although in stem cells telomerase is active to ensure the maintenance of telomere length (Greider and Blackburn, 1989; Feng et al., 1995; Nakamura and Cech, 1998). In hESCs, the telomerase enzyme is continually active in order to maintain the extended length of telomeres and in iPSCs, telomerase is re-activated after reprogramming and the process of telomere lengthening begins (Takahashi and Yamanaka, 2006; Takahashi et al., 2007; Marión et al., 2009). When two end-to-end fused chromosomes are being pulled apart by opposing mitotic spindle tubules, anaphase bridges or chromatin bridges can occur which create a link between the two daughter cells. Although the formation of anaphase bridges does occur in normal cells (Baumann et al., 2007; Chan et al., 2007), it is strongly associated with the erosion of telomeres (Tusell et al., 2010). The inability of the fused chromosomes to part leads to one daughter cell gaining a chromosome and the other losing a chromosome. Further, end-to-end fusion of chromosomes can cause breakage-fusion-bridge (BRB) cycles to be established, resulting in genomic instability (DePinho, 2000; Gisselsson et al., 2001; Hackett et al., 2001) and in turn causing the shearing of ultra-fine bridges also generating aneuploidy.

Telomeric sequences are associated with a group of proteins; TRF1, TRF2, RAP1, POT1, TIN1, and TIN2, collectively known as the shelterin complex (Liu et al., 2004). Disruption of these proteins can cause fragile sites in the genome, contributing to DNA replication defects (Sfeir et al., 2009), anaphase bridges (Bunch et al., 2005; Nera et al., 2015), chromosome fusions (Pardo and Marcand, 2005) and the activation of DNA damage responses (Palm and de Lange, 2008). A recent study has revealed that overexpression of the telomere repeat-binding factor 1 (TRF1) in mouse ESCs can indeed cause anaphase bridges to form (Lisaingo et al., 2014), thus indicating the importance of telomere protection in hESCs. Most interestingly, in ESCs with short telomeres (Huang et al., 2011) and in the full knockout of a subunit of telomerase, Tert -/- ESCs (Pucci et al., 2013), reduced levels of pluripotency have been observed. Indeed, long telomeres and high TRF1 levels have been proposed as additional stem cell markers (Flores et al., 2008; Huang et al., 2011; Schneider et al., 2013). However, although the overexpression of telomerase did improve the self-renewal and proliferation rate, it increased resistance to apoptosis and caused a suppression in the differentiation capacity of ESCs (Armstrong et al., 2005; Yang C. et al., 2008). These findings suggest a potential range for optimal telomere length in the hPSCs, which could be used as a screening method, in the cells intended for clinical use.

On occasion, the sister chromatids are not resolved correctly during mitosis, due to the lack of kinetochore attachment to the mitotic spindle, with one daughter cell receiving both chromosomes, and an aneuploid status in both cells. How the mitotic spindles assemble in hPSCs is not well investigated, however, spindle defects such as asymmetric orientation have been linked with carcinogenesis in *Drosophila melanogaster* (Caussinus and Gonzalez, 2005; Castellanos et al., 2008) and in human gut epithelial stem cells (Quyn et al., 2010). A balance of symmetric or asymmetric cell divisions are necessary for normal development and tissue homeostasis, however this can lead to abnormal proliferation (Noatynska et al., 2012). Alternatively, lagging chromosomes derived from mitotic spindle detachment or the bipolar orientation of chromatids (Cimini et al., 2002) can instead form a separate compartment of chromatin away from nuclei. Atelometric and acentric, whole or fragmented chromosomes, can become micronuclei (Cimini et al., 1999; Minissi et al., 1999; Norppa and Falck, 2003) or double-minute (DM) chromatin, where small fragments of amplified genes occur extra-chromosomally (Haaf and Schmid, 1988; Itoh and Shimizu, 1998). Although nuclear contents may be lost in this manner, they can also be engulfed into nuclei (Minissi et al., 1999). Micronuclei or DMs can appear as a result of replicative stress and sometimes still remain transcriptionally active, albeit at reduced levels (Hoffelder et al., 2004; Utani et al., 2007). These micronuclei can also contain nucleoskeletal structural components such as nuclear lamins and thus are not totally inert (Tanaka and Shimizu, 2000). Both pluripotent and differentiating ESCs seem to have a propensity to form micronuclei: in mouse ESCs, an increase in micronuclei formation and apoptosis was observed with the downregulation of the pluripotency marker *OCT4* (Zhao et al., 2014), additionally differentiation of murine ESCs to neural progenitor cells causes a nearly 2-fold increase in micronuclei formation and an increase in chromosome instability (Sartore et al., 2011). Indeed, the high rate of proliferation of hESCs in itself could promote the formation of micronuclei and thus be a factor contributing to their genomic instability (Stopper et al., 2003).

The apoptosis inhibitor protein, survivin, normally protects against polyploidy through its function in the control of the spindle assembly checkpoint and cytokinesis. Impairment of survivin expression has been associated with polyploidy development in human cells (Li et al., 1999). Survivin is highly expressed in ESCs (Adida et al., 1998) and has been shown to be fundamental in maintaining pluripotency (Mull et al., 2014; Kapinas et al., 2015) by being involved, with its splice variants, in the upregulation of *NANOG* and *OCT4* (Mull et al., 2014). Thus, there is a case for survivin expression to be tested for as part of a genomic health screen for clinical-grade stem cells.

### DNA Damage

During development, blastocyst cells may have to compromise their DNA proof-reading capability in order to achieve a rapid rate of cell division. This postulation is supported by the shortened G_1_ phase of interphase in ESCs in culture (Becker et al., 2006; Ghule et al., 2008), exposing them to potentially higher replicative errors. Furthermore, studies of the TP53-p21 pathways in hESCs have revealed that during stress stimuli, the p21 mRNA is upregulated in hESCs, however no p21 protein is detected (Dolezalova et al., 2012). This could imply that although the cell has responded to stress, it has not been able to achieve p21 function, allowing replication errors to remain. During DNA damage in hESCs, TP53 binds directly to *NANOG'*s promoter, suppressing it and promoting hESC differentiation (Lin et al., 2005). If p53 levels are reduced, the levels of spontaneous differentiation are also reduced (Kawamura et al., 2009). It seems that in hiPSCs, DNA damage does not give rise to single-stranded DNA regions, checkpoints are not activated, and thus DNA repair does not occur (Desmarais et al., 2012), despite there being elevated expression levels of DNA repair genes (Momcilovic et al., 2010). This is echoed in studies of mouse cells, whereby iPSCs were less able to perform double-strand break repair, especially by homologous recombination repair, compared with both primary cells and ESCs (Zhang et al., 2018). Furthermore, hiPSCs have been found to be deficient in intra-S checkpoints and also in G_2_/M decatenation or chromatin dis-entanglement, preventing delayed entry of inappropriately condensed chromosomes into mitosis and permitting the formation of anaphase bridges (Damelin et al., 2005; Filion et al., 2009; Weissbein et al., 2014; Lamm et al., 2016). Topoisomerase II permits chromatin decatenation to occur in G_2_ to delay mitosis and allow smooth sister chromatid segregation (Uemura et al., 1987; Holm et al., 1989). When the decatenation checkpoint is disrupted, entangled chromosomes segregate and then form new cells with aneuploidy (Gorbsky, 1994; Andoh and Ishida, 1998). Chromosome decatenation deficiency has also been reported in mouse ESCs and human multipotent progenitor cells, however improved decatenation was observed later with cell differentiation (Damelin et al., 2005). The reason behind such entanglement of ESC chromatin may be due to the lack of higher chromatin organization in the nucleus, such as heterochromatin. hESC nuclei lack chromatin silencing markers, such as methylation on H3K9 and H3K27. The plasticity of the chromatin, causes the DNA to be a highly open structure and coupled with the dispersed presence of the DNA damage marker, γ-H2AX in hESCs (Meshorer et al., 2006), in stark comparison to more localized foci in somatic cells (Mariotti et al., 2013), suggests a more exposed, and therefore a more easily damaged chromatin. The plasticity of the more-open chromatin state in stem cells could be one of the reasons for the increased genomic instability of hPSCs when cultured *in vitro*. Increased levels of γ-H2AX were also noted in hiPSCs compared with their source primary line (Vallabhaneni et al., 2018), suggesting a similar scenario in these cells. Although, this may be debatable since no additional protection of heterochromatin, in comparison to euchromatin, has been observed from the reactive oxygen species (ROS)-induction of double-stranded breaks (Woodbine et al., 2011). But, lower levels of Ataxia-telangiectasia mutated kinase (ATM) phosphorylation in iPSCs has been previously reported in cells treated with low levels of radiation, alongside hypersensitivity to apoptosis (Nagaria et al., 2016). ATM phosphorylates a number of proteins, related to apoptosis, cell cycle checkpoints, and DNA repair (Lee and Paull, 2007), therefore its potentially reduced role in hPSCs should be carefully considered. The exact role of ATM in DNA damage in heterochromatin is still unknown, but it has been suggested to be preferentially required in the DNA damage repair of heterochromatin (Goodarzi et al., 2008). As hPSCs lack the presence of heterochromatin (Francastel et al., 2000; Meshorer and Misteli, 2006), the reduced levels of ATM phosphorylation (Nagaria et al., 2016) probably would not have a significant effect on the genomic integrity of the cell. However, ATM-deficient cells were less efficient in reprogramming to iPSC, which influenced the appearance of genomic variation (Marión et al., 2009; Kinoshita et al., 2011; Lu et al., 2016). Similarly, Artemis, an endonuclease associated with non-homologous end-joining, is required for the maintenance of genomic stability (Woodbine et al., 2011), but its absence from stem cells did not impair myeloid differentiation, reprogramming or show any signs of significant genomic instability (Felgentreff et al., 2014).

Despite the susceptibility of hPSCs to DNA damage *in vitro*, steps may be taken to alleviate this by the modification of culture conditions, including freeze-thaw techniques, passaging (Mitalipova et al., 2005), and media composition: a reduction in MEK inhibition (involved in the regulation of DNA damage/repair and cell cycle) was observed to maintain naive hESCs, accelerate proliferation, and reduce the accumulation of chromosomal abnormalities in culture (Di Stefano et al., 2018).

### Bystander Effect?

Another putative mechanism for the process of aneuploidy accumulation is that cells acquire an aneuploidy and then via a bystander effect further aneuploidies accumulate in neighboring cells. Such mechanisms have been observed with radiation-treated cells causing cell senescence in neighboring cells (Nelson et al., 2012), increased sister chromatid exchange (Nagasawa and Little, 1992; Deshpande et al., 1996), increased *TP53* expression (Hickman et al., 1994; Azzam et al., 1998), and most importantly chromosomal instability (Lorimore et al., 1998; Sawant et al., 2001). This instability in the irradiated cells is probably observed due to the ROS produced from the radiation (Yamamori et al., 2012) causing DNA damage to occur (Yermilov et al., 1996; Balasubramanian et al., 1998). Most interestingly, a bacterium species has been shown to induce aneuploidy, amongst other hallmarks of genomic instability, in human cells, through a bystander effect. *Enterococcus faecalis*, an intestinal bacterium, where the production of ROS molecules induced chromosome instability in cells with defects in mismatch repair genes (Huycke et al., 2001, 2002; Wang et al., 2008). Although this theory needs to be investigated further, it is well established that ROS and nitrogen species from both radiation and metabolism can cause oxidative stress that can lead to DNA damage and senescence in cells (Lindahl, 1993; Suh et al., 1999; Geiszt et al., 2000). Moreover, it may be the case with hPSCs that if one event triggers an aneuploidy to occur, a bystander effect could then cause neighboring cells to also acquire aneuploidies, through transmission of substances through the culture media or delivered in exosomes. For example, if mitomycin C, a commonly used growth inhibitor of feeder cells, were to negatively affect the hPSC basement membrane, then we theorize that this might affect the neighboring stem cells. This event can then cause or promote the generation of further aneuploidies in the hPSC culture. As more hESC lines are developed on, or adapted to other alternative matrices, it should become more apparent if there are any effects and whether it is the stem cells or the feeder cells that potentially instigate aneuploidy.

It has been previously proposed that the increased age of cells and the amount of ROS are linked (Finkel and Holbrook, 2000). As human pluripotent stem cells are metabolically very active and can be maintained in cultures for long periods of time, the increased age and the fast metabolism required in these cells could also be an aspect that factors in the genomic instability often observed. In contrast, it has been reported that both high and low levels of ROS can impair the reprogramming ability of cells into iPSCs (Zhou et al., 2016) and elevated levels can impair their differentiation ability as well (Rönn et al., 2017). These studies suggest that optimal levels of ROS may be required for the cells to grow stably in culture. With the effect of ROS established above, very precise growth conditions must be maintained in the hPSC culture to ensure genomic integrity. We hypothesize that the use of reagents, such as mitomycin C, could potentially affect the neighboring hPSCs and should be carefully considered before the assumption of no effect.

### Nuclear Lamin Depletion

Lamins are a meshwork of proteins found at the nuclear periphery with intimate associations with the inner nuclear membrane and co-located proteins (Gruenbaum et al., 2000; Zastrow et al., 2004). Nuclear lamins, which play an important role in the maintenance of nuclear morphology and chromosome organization (Aebi et al., 1986; Bridger et al., 2007; Dechat et al., 2008; Bickmore and van Steensel, 2013), have also been suggested to be involved in many other processes within the nucleus, such as DNA replication and repair, transcription and RNA processing (Cai, 2001; Laguri et al., 2001; Wolff et al., 2001; Spann et al., 2002).

In humans, A-type lamins, such as lamin A and C, are encoded by *LMNA*, whereas B-type lamins, such as lamins B1 and B2 are encoded by *LMNB1* and *LMNB2*, respectively (Wydner et al., 1996). Unlike A-type lamins, lamins B1 and B2 are endogenously expressed in both somatic and embryonic cells (Höger et al., 1990; Pollard et al., 1990; Lin and Worman, 1995). The presence of A-type lamins in embryonic cells is still debated, as some reports show that A-type lamins are expressed only in somatic cells (Lehner et al., 1987; Stewart and Burke, 1987; Höger et al., 1990; Hutchison, 2002), and are completely absent from the nuclei in both ESCs (Constantinescu et al., 2006) and iPSCs (Mattout et al., 2011), whereas more recent reports suggest that A-type lamins are expressed at low levels in ESCs (Kim et al., 2011; Eckersley-Maslin et al., 2013). In early embryos, A-type lamins can be observed (Foster et al., 2005), but these are thought to be gamete-derived and soon disappear.

A-type lamins are found to accumulate with the down-regulation of *OCT4*, a hallmark of cell differentiation, and this is thought to contribute to the ESC nuclear plasticity (Constantinescu et al., 2006; Meshorer et al., 2006; Pajerowski et al., 2007). Lamin A then associates with and anchors, forming heterochromatin at the nuclear periphery, helping to organize the genome, regulating it for lineage commitment (Solovei et al., 2013); the accumulation of A-type lamins during differentiation have been associated with the loss of nuclear plasticity (Constantinescu et al., 2006; Meshorer et al., 2006; Pajerowski et al., 2007). Mutations in the A-type lamins give rise to a family of diseases commonly referred to as laminopathies, often associated with tissues derived from the mesenchyme, such as skeletal muscle, skin, cardiac muscle, tendons, adipose, and neurons (Worman and Bonne, 2007). Indeed, *LMNA* mutations cause impaired differentiation of adult mesenchymal stem cells (Gotzmann and Foisner, 2006; Pekovic and Hutchison, 2008; Scaffidi and Misteli, 2008), alterations in *Notch* and *Wnt* signaling pathways required for early development (Espada et al., 2008; Meshorer and Gruenbaum, 2008; Scaffidi and Misteli, 2008; Hernandez et al., 2010) and MSC death (Halaschek-Wiener and Brooks-Wilson, 2007; Meshorer and Gruenbaum, 2008; Prokocimer et al., 2009). Additionally, lamin A knockdown affects the serum response factor (SRF) pathway that promotes expression of abundant actin-myosin cytoskeletal components involved in the differentiation of cells (Swift and Discher, 2014). The SRF pathway is partially regulated by nuclear actin (Olson and Nordheim, 2010; Baarlink et al., 2013), which binds to lamin A (Simon et al., 2010) and other proteins associated with lamin A, such as emerin (Simon and Wilson, 2011). In contrast, Lamin B1 and B2 knockout does not affect the differentiation of blastocysts, but does affect organogenesis in mice (Coffinier et al., 2010; Kim et al., 2011), as well as mitotic spindle orientation and formation (Tsai et al., 2006; Ma et al., 2009; Kim et al., 2011). This suggests that B-type lamins have a functional role in ensuring chromosomes are efficiently segregated during mitosis. This correlates with findings of lamin B2 depletion being associated with aneuploidy formation, prolonged mitosis and formation of anaphase bridges in cancerous cells (Kuga et al., 2014; Ranade et al., 2017). Additionally, the depletion of lamin B2 caused the mislocalization of chromosome territories (CTs) in aneuploid cells (Ranade et al., 2017). In contrast, in mouse ESCs the knock-out of B-type lamins and the mutation of *Lmna* did not cause any effect on the proliferation and differentiation of mouse ESCs, nor did it change the total number of chromosomes in nuclei (Kim et al., 2013). It has been suggested that lamin B2, alongside the inner nuclear membrane protein SUN1 (Malone et al., 2003; Razafsky and Hodzic, 2009), supports the spindle pole during mitotic spindle formation (Kuga et al., 2014). Indeed, SUN1 is required for telomere binding to the nuclear envelope and disruption of SUN1 affects meiotic division (Ding et al., 2007). We hypothesize that nuclear proteins, especially lamins, have a key role in the maintenance of genomic stability of hPSCs. Further work is required to establish whether B-type lamin loss causes aneuploidies or aneuploidies induce the loss of B-type lamins.

### Chromosome Integrity Checkpoints

With all the scenarios that can go wrong in a cell with respect to genomic instability, chromosome integrity and DNA damage it is important that cells have adequate and well-functioning checkpoints, to assess the health of the genome (Sperka et al., 2012). For correct chromosome segregation there are two critical checkpoints, known as the spindle assembly checkpoint and the decatenation checkpoint. The G1 tetraploidy checkpoint also assesses for chromosome aberration, especially additional chromosomes (Brown and Geiger, 2018). Very interestingly in murine ESCs the spindle assembly checkpoint was not activated as it would be in somatic cells, leading to apoptosis and so the possibility of a higher numbers of cells with aneuploidy (Rohrabaugh et al., 2008). Furthermore, the decatenation checkpoint which verifies for entanglement of chromosomes that can happen with inadequate DNA damage repair, has been revealed to not be activated in murine ESCs, although it is activated once cells have committed to a lineage (Damelin et al., 2005; Suvorova et al., 2016). Thus, the lack of checkpoint function in embryonic stem cells is perhaps a process to maintain stemness and openness of chromatin, allowing aneuploidy and instability to arise in a population but which can be overcome later, removing individual cells that are too compromised. A further checkpoint that monitors the numbers of centrosomes, a building block of the spindle pole bodies has not yet been studied in stem cells; such a screening test to assess centrosome number by antibody staining probably should be included in a panel of assessments and parameters to be tested prior to stem cell use in the clinic.

**Figure 2:**
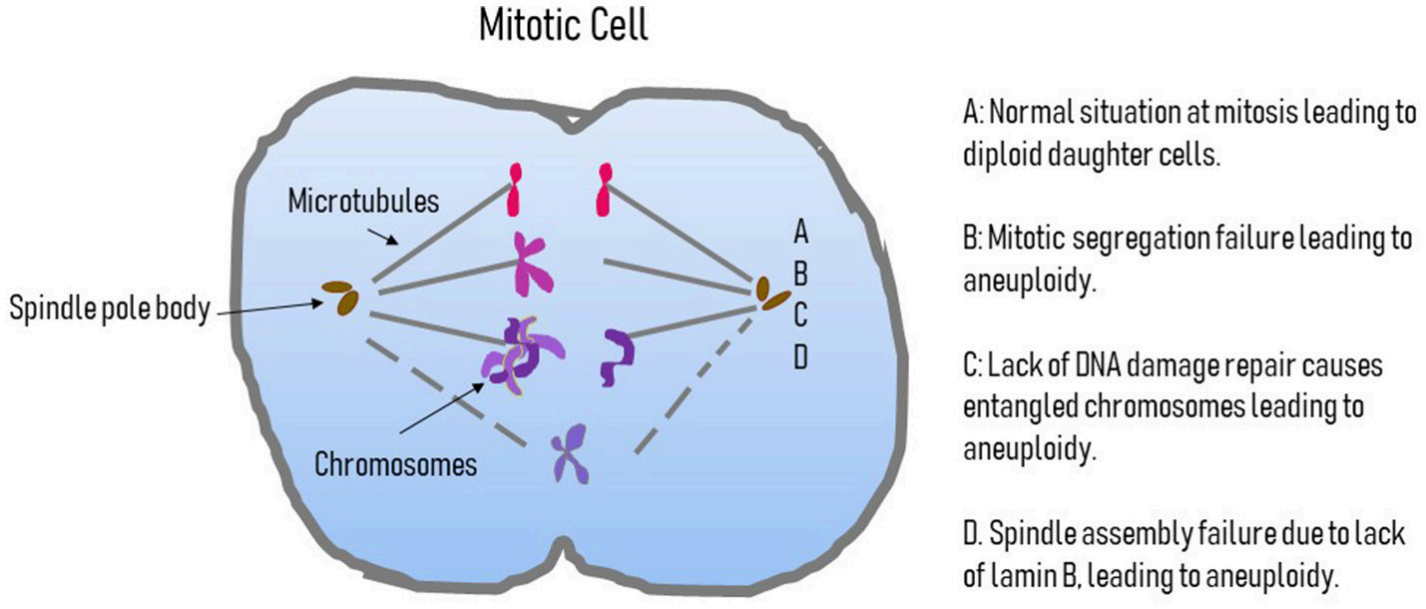
Possible causes of aneuploidy in pluripotent cells. This figure displays a cartoon of a mitotic cells outlining the possible causes of aneuploidy. A is the normal situation where the centromere attaches to the microtubules of the spindle and a normal segregation occurs. B highlights a failure of segregation where the chromosomes do not divide and an extra copy of a chromosome will be in one daughter nucleus and missing in the other. C is the situation where DNA damage is not repaired properly and leads to entangled chromosomes that cannot segregate correctly, again giving an additional chromosome in one daughter nucleus and a lack of that chromosome in the other. D represents the situation where issues with the complement of B-type lamins, specifically B2, leads to spindle assembly failure and so chromosomes are lost or non-segregated chromosomes can become encompassed into one of the reforming daughter nuclei.

Cyclin D1 levels are low in ESCs as compared to somatic differentiated cells. Cyclin D1 is a pivotal component of the G1/S transition in interphase. Interestingly, it is the presence of specific microRNAs regulated by *OCT4* and *SOX2* that prevent the expression of cyclin D1 (Card et al., 2008). For iPSCs, reprogramming back to a less controlled cell cycle, with “looser” checkpoints and shorter G1 and G2 phases is thwarted by cyclin D1 (Chen et al., 2014). Figure 2 gives an overview of the causes discussed that may permit aneuploidy to arise.

### Genome Organization Is Different in Stem Cells

Earlier studies have analyzed the genome in somatic and indeed stem cells with specific chromosome probes in fluorescence *in situ* hybridization (FISH) visualized by high resolution microscopy (Clements et al., 2016). The genome is highly organized in somatic, differentiated cells (Bridger and Bickmore, 1998; Parada and Misteli, 2002; Tanabe et al., 2002; Foster et al., 2012), with interphase chromosomes organized into individual territories (Cremer and Cremer, 2001), called chromosome territories in similar nuclear locations between different cell types, with a few specific tissue related differences (Kuroda et al., 2004; Parada et al., 2004; Foster et al., 2012; Robson et al., 2016). On the whole, in proliferating cells a gene-density distribution is observed with gene-rich chromosomes found toward to the nuclear interior and gene-poor toward the nuclear periphery (Bridger et al., 2014). A re-positioning occurs when cells leave the proliferative cell cycle to quiescence or senescence (Bridger et al., 2000; Mehta et al., 2010; Criscione et al., 2016). Here, we review how chromosomes are arranged in hPSCs compared with somatic cells and discuss whether the type of strict genome organization and chromosome positioning found in differentiated cells is pertinent and relevant to stem cells.

A gene-density radial distribution of CTs has been observed in hESCs (Wiblin et al., 2005; Bártová et al., 2008), as it has been in human somatic cells (Croft et al., 1999; Boyle et al., 2001) and in human blastomeres (Finch et al., 2008). These data were corroborated for stem cells by studies in pig cells whereby there was very little difference in chromosome positioning between mesenchymal stem cells from bone marrow and cells within differentiated tissues (Foster et al., 2012). However, gene-rich human chromosomes 17 and 19 were positioned more centrally in granulocytes when compared to hESC (Bártová et al., 2001), even though chromosome 12 and its centromere positioning in pluripotent and somatic cells were reportedly the same (Bártová et al., 2008). These data indicate that CT positioning in ESCs is not as it will be once the cells have differentiated. This would suggest that embryonic nuclei have mechanisms in place to re-position interphase chromosomes. Further, in cloned bovine embryos, CTs also do not relocate upon development but the pluripotency genes are relocated to more transcriptionally active regions of the territories (Orsztynowicz et al., 2017). Genes looping away from CTs has been reported previously to be associated with dependent transcription in specific cell types (Volpi et al., 2000; Mahy et al., 2002). Indeed, the 12p region that contains a group of clustered pluripotency genes, including *NANOG*, was found to be located more centrally in hESCs than in somatic cells (Wiblin et al., 2005). In contrast, chromosome 6p, containing the pluripotency marker *OCT4*, did not show any difference in its nuclear position, whilst the *OCT4* locus was reported to move to outside its CT in ESCs (Wiblin et al., 2005).

Reports of a less rigid chromatin state, due in part to the lack and/or absence of chromatin remodeling markers, in undifferentiated cells has been reported (Keohane et al., 1996; Francastel et al., 2000; Lee et al., 2004; Meshorer et al., 2006). In normal somatic cells, centromeres are mostly found nearer to the nuclear periphery or around nucleoli, and also often by the CT periphery (Weierich et al., 2003; Gilchrist et al., 2004), although this may depend on the stage of the cell cycle (Ferguson et al., 1992; Weimer et al., 1992; Hulspas et al., 1994). Previous reports have found that in human cells during differentiation, centromeres tend to move nearer to the nuclear periphery (Salníková et al., 2000; Bártová et al., 2001; Galiová et al., 2004; Horáková et al., 2010), or relocate more centrally (Bártová et al., 2008) to the heterochromatin surrounding nucleoli, and cluster together in chromo-centromeres (Alcobia et al., 2000; Beil et al., 2002). Movement of the centromeres toward the nuclear periphery was also observed in early rabbit embryos, once they had passed the 4-cell stage (Bonnet-Garnier et al., 2018). Such heterochromatic chromosomal regions may be more likely to be positioned toward the nuclear periphery which is supported by the findings of an increased association of chromatin silencing markers with perinuclear centromeres (Bártová et al., 2008) and with the under-acetylation of centromeres in both mouse and human undifferentiated cells (O'Neill and Turner, 1995; Keohane et al., 1996). Immaturely developed centromeres, lacking specific markers of heterochromatin, in embryos and stem cells might be less able to attach to the mitotic spindle, resulting in aneuploidy. Indeed, interfering with centromere structure does lead to mitotic catastrophe in mice (Howman et al., 2000; Artus et al., 2006).

**Figure 3:**
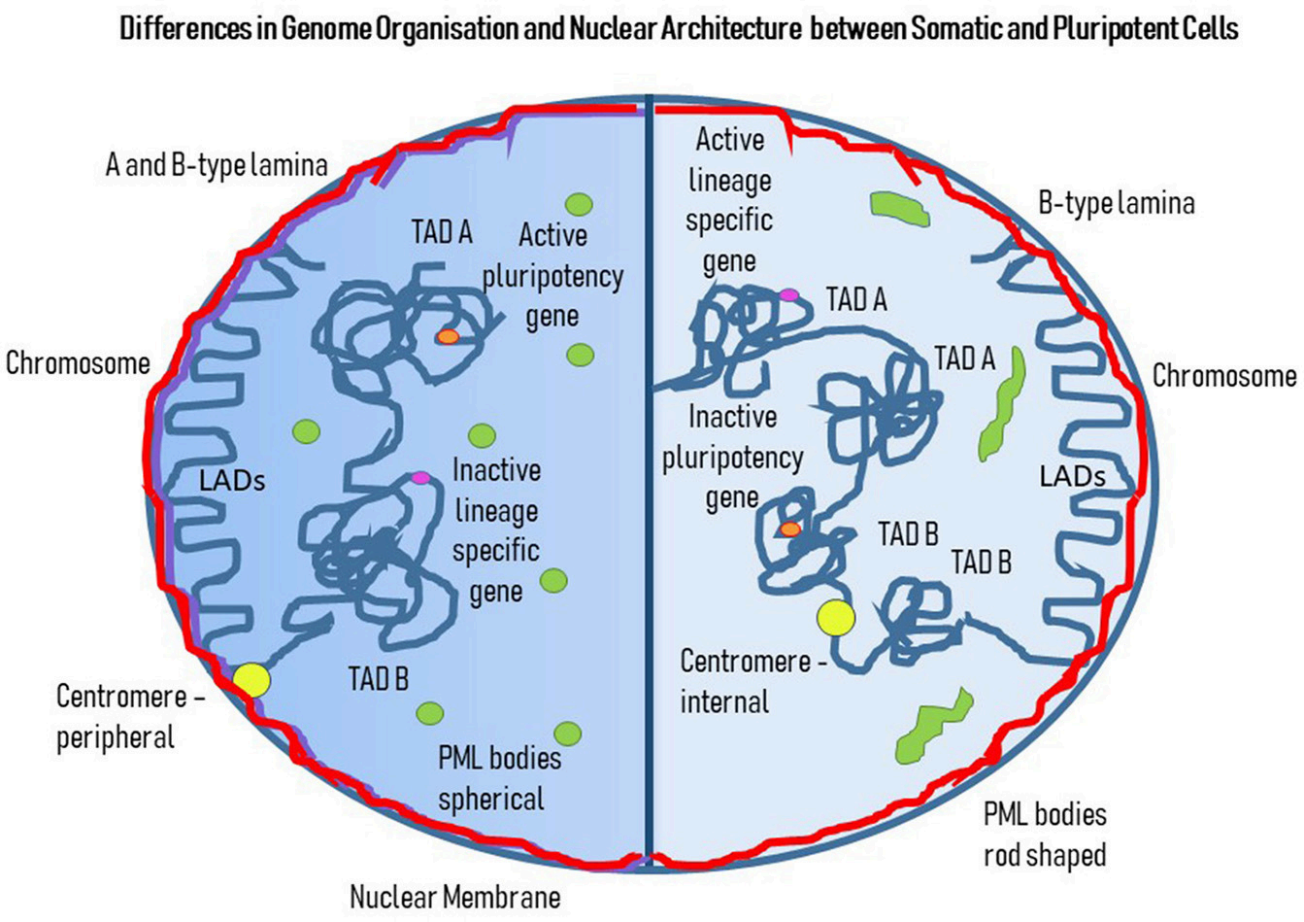
Differences in Genome Organization and Nuclear Architecture between Somatic and Pluripotent Cells. This cartoon displays a cell with two halves. The darker left hand side represents genome organization and nuclear architecture in a somatic cell and the right hand half is a pluripotent cell. The nuclear lamina subjacent to the nuclear membrane represents a mixture of A (purple) and B-type (red) lamins, whereas in the PSC there are only B-type lamins. The PML bodies (green) have a different shape and position in the somatic cells compared to the PSC; in the somatic cell they are spherical and found throughout the nucleoplasm, whereas in the PSC they are elongated rods in shape and are found more toward the nuclear edge. Concerning the genome, there are both LADs and TADs, with LADs looking very similar between the somatic and pluripotent cells, whereas there are more TADs of a smaller size in PSC compared to the somatic cell. Pluripotency genes are active (orange) and found in A-type TADs in PSCs but are inactivated and found in B-type TADs in somatic cells. Lineage specific genes (pink) are shut-down in PSCs but activated in somatic cells, with association with B TADs and A TADs, respectively. Centromeres (yellow) are more peripheral in somatic cells whereas in PSCs they can be found more internally.

More recently global genome organization has been analyzed by a range of chromosome conformation capture (3C) experiments. Based on forming cross-links between pieces of chromatin that sit adjacent to each other, fragmenting, ligating and sequencing the new ligated DNA pieces reveals which parts of the genome sit together in three-dimensional space within nuclei. These studies have revealed that the genome is folded and organized into topologically associated domains (TADs) which have two sub-types A and B (Lieberman-Aiden et al., 2009; Dixon et al., 2012; Nora et al., 2012; Sexton et al., 2012). Type A TADs contain active open chromatin whereas B-type TADs are comprised of inactive more heterochromatic regions of the genome (Figure 3). These TADs have been found not only in somatic cells but in ESCs too, revealing similar types of organization of the genome present before differentiation. However, in ESCs the number of TADs are increased and the size is reduced, suggesting that there is in fact a less organized genome organization (Glinsky et al., 2018). However, closer study with 3C combined with chromatin factor binding data reveal that inactive chromatin in PSCs is not organized as would be expected in somatic cells (de Wit et al., 2013) and there is noticeably less heterochromatin. Whereas, active regions of the genome bound by pluripotency factors such as NANOG and OCT4 bring specific clusters of genes together (de Wit et al., 2013) to maintain pluripotency. Indeed, at the *NANOG* locus, specific proteins interact to regulate NANOG expression being bound together in an “interactome” containing mediator, a transcriptional coactivator and a chromosomal architectural protein with cohesin with the other key players in pluripotency *SOX2*, c-*MYC*, and *OCT4* (Apostolou et al., 2013). Others have shown that OCT4 behaves in a similar way in mouse and humans iPSC construction (Wei et al., 2013; Zhang et al., 2013). Phillips-Cremins discusses the differences in ESC nuclei with respect to gene association with the different TAD sub-types and how this can switch upon differentiation (Phillips-Cremins, 2014). Indeed, pluripotency genes move from associating with A TADs to B TADs (Lin et al., 2012). The association of the genome with the nuclear periphery is also massively altered in mouse ESCs with genes required to maintain pluripotency away from the repressive environment of the nuclear edge (Peric-Hupkes et al., 2010). Figure 3 gives an overview of the differences between ESCs, iPSCs, and somatic cells, with respect to genome organization.

It is as yet not clear the effect that aneuploidy could have on genome organization, with extra genomic regions needing space at the nuclear envelope or elsewhere. Indeed, although reports show that extra chromosomes are located in the correct nuclear compartment in somatic cells, the same is not as clear for pluripotent cells that lack A-type lamins and have other altered nuclear architecture. Gene expression can be changed on a large scale when there are extra chromosomes, and this could be a more important issue than more simply having extra copies of some genes. Thus, the real impact of extra chromosomes on genome organization into TADs and indeed lamina-associated domains (LADs, see below) and genome function as a whole remains to be elucidated.

### Nuclear Architecture and Sub-Components

The nuclear lamina is located at the nuclear envelope and is comprised of A and B–type lamins, combined with a plethora of nuclear envelope transmembrane proteins (Czapiewski et al., 2016) with many of these proteins having chromatin binding abilities. Indeed, the nuclear lamins are chromatin-binder and anchoring specific regions of the genome through LADs (van Steensel and Belmont, 2017). LADs are regions of the genome that on the whole are comprised of heterochromatin and repressed sequences. This is not the case for genes that are more proximal to nuclear pore complexes that can be active. In mouse and human iPSCs, LADs have a higher mutation rate than in non-LADs which could be due to oxidative stress generated during the reprogramming process (Yoshihara et al., 2017) (Figure 3).

In human and mouse ES cells, the presence of lamins B1 and B2 was observed with lamin A/C absent (Constantinescu et al., 2006). Removal of lamin B1 in murine ESCs appeared in one study to be essential for heterochromatin to be located at the nuclear periphery (Zheng et al., 2015) but in another study, the lack of all nuclear lamins, both A-type and B-type did not have any effect on genome organization and LAD positioning, implying that other proteins are responsible for the positioning and anchorage of chromatin through LADs at the nuclear envelope, for example the integral membrane protein emerin (Amendola and van Steensel, 2014). In another study, Robson et al. demonstrated how nuclear envelope transmembrane proteins NET39, TMEM38A, and WFS1 anchor myogenic specific genes to the nuclear periphery for repression in stem cells prior to differentiation (Robson et al., 2016). Despite some studies (Eckersley-Maslin et al., 2013), the A-type lamins do not appear to be expressed or required by undifferentiated embryonic stem cells (Rober et al., 1989; Smith et al., 2017) and also have been observed to completely disappear with successful reprogramming of iPSCs (Mattout et al., 2011; Zuo et al., 2012). Indeed, it seems that A-type lamin upregulation is concomitant with or even responsible for the start of lineage commitment. The incorporation of A-type lamins and emerin into the nuclear lamina induces size and morphology changes in nuclei (Butler et al., 2009), and correspondingly, nuclei lacking A-type lamins and emerin fail to change their morphology, with compromised ability to undergo endoderm differentiation, along with changes in gene expression (Smith et al., 2017). A-type lamins were also found to accumulate with the downregulation of *OCT4*, a hallmark of differentiation. The absence of lamins A/C has been suggested to contribute to the ESC nuclei plasticity compared to the more rigid state of somatic cell nuclei, with hESC lacking heterochromatin at the nuclear periphery (Smith et al., 2017) and a global remodeling of the genome organization during lineage commitment (Peric-Hupkes and van Steensel, 2010). Mutations in the lamin A gene, *LMNA*, that cause muscular dystrophy, interfered with the formation of typical LADs at the nuclear envelope, altering their heterochromatic status which as a consequence changed the repression of the *SOX2* locus, allowing them to be upregulated (Perovanovic et al., 2016). Lamin A knockdown affects the SRF pathway that promotes expression of abundant actin-myosin cytoskeletal components involved in the differentiation of cells (Swift and Discher, 2014). The SRF pathway is partially regulated by nuclear actin (Olson and Nordheim, 2010; Baarlink et al., 2013), which binds to lamin A (Simon et al., 2010) and other proteins associated with lamin A, such as emerin. This would suggest a functional role of lamin A in the indirect regulation of the differentiation of cells via an inhibitory effect on nuclear actin and myosins. Nuclear actin and myosin have been shown to work in concert to move regions of the genome around nuclei (Fedorova and Zink, 2008; Mehta et al., 2010; Bridger and Mehta, 2011; Kulashreshtha et al., 2016), but they are also involved in gene expression and processing. With the significant changes at the nuclear lamina between the pluripotent state and the somatic/lineage situation it seems unlikely that there are not changes with respect to LADs associating with the nuclear lamina, even though they have not been revealed. Indeed, LADs can also be internally located near A-type lamins (Briand et al., 2018) and so genome organization would be expected to change substantially after the A-type lamins arrive (Figure 3).

### Promyelocytic Leukemia Bodies

There exists an emerging role for promyelocytic myeloid (PML) bodies in stem cell pluripotency and reprogramming, with their presence required to maintain pluripotency and reprogramming of cells to iPSCs (Hadjimichael et al., 2017). Some regard PML bodies in hESCs as comparable structures to those in somatic cells (Wiblin et al., 2005; Meshorer and Misteli, 2006), with their spherical unmistakeable morphology. Alternatively, one study argues that PML bodies in stem cells and somatic cells are long linear structures or “rods and rosettes” in the embryonic stem cell nuclei. The study suggested that the unique PML bodies appear in the early stages of the cell life before any epigenetic imprinting may occur. Unlike in somatic cells, the PML bodies would often associate with the nuclear edge and appear less frequently, independent of different cell line, feeder/matrix, passaging method and the stage of cell-cycle (Butler et al., 2009). Additionally, the “rods and rosettes” were often found to appear near the edge of the undifferentiated ESC colonies. Additionally, Lawrence and colleagues (Butler et al., 2009) found that the composition of the PML bodies is different to that found in somatic cells. hESC PML bodies were found to not contain SUMO, SP100, or DAXX, which are usually present in those of somatic cells. These findings have been supported by Tokunaga et al. (2014), who have also found similar “rod” structures in their reprogrammed iPSCs. Additionally, it was suggested that the round “rosettes” found in their reprogrammed cells that failed to produce successful iPSCs was a sign of a transitional stage from somatic cell to iPSC (Tokunaga et al., 2014). Salsman et al. revealed PML body loss upon differentiation of myoblasts and the relocation of DAXX protein (Salsman et al., 2017) (Figure 3).

The question concerning the differences in genome organization in ESCs and iPSCs is whether it is important to assess with respect to risk in a whole organism? It seems that genome organization is more dis-organized and plastic and possibly more random. But whether this is detrimental is debatable since there is evidence that once cells have initiated their lineage journey these aspects are corrected. However, there may be more genome instability evident and the consequences that follow such a situation i.e., chromosomal aberrations. This may be the downside of maintaining a plastic open genome and the question as to whether an adult, possibly of advanced age, has the same capacity to tolerate genomically compromised cells remains.

### Epigenetic Modifications in Pluripotent Cells

How exactly specific chromatin conformation in ESC nuclei influences differentiation is unknown, however there has to be a certain openness of the chromatin (Meshorer and Misteli, 2006), with markers such as H3K4me3 (Harikumar and Meshorer, 2015). Presumably, this flexibility permits a normal global gene activity in the cells, whilst cells remain pluripotent and maintain their self-renewal capacity. This theory is supported by findings of an increased accumulation of heterochromatin upon differentiation (Francastel et al., 2000), implying that with a reduced need of certain genes in specific cell types, transcription can be silenced (Jiménez et al., 1992; Hu et al., 1997). The chromatin state of terminally differentiated cell types is more “rigid,” in comparison to cells with differentiation capability (Meshorer et al., 2006). This would be an efficient way to establish tissue-specific gene expression and has been found to be true for the differentiation of mammalian hemopoetic cells and in *Caenorhabditis elegans*; with more terminally differentiated cells having more heterochromatin accumulation (Reviewed in Francastel et al., 2000). Indeed, differentiation-dependent chromatin modifications are observed with an increase of silencing chromatin markers, such as H3K9me3 and global cytosine methylation (Lee et al., 2004; Meshorer et al., 2006), decreased active chromatin markers, such as H3K4me3 (Guenther et al., 2010) and increased H4 deacetylation in centromere heterochromatin as cells differentiate (O'Neill and Turner, 1995; Keohane et al., 1996). Interestingly, in hESCs many genes show both chromatin marks; for repression H3K27me3 and for expression H3K27ac and H3K4me3, indicating genes are poised ready for expression once differentiation is initiated (Harikumar and Meshorer, 2015; Theunissen and Jaenisch, 2017; Godini and Fallahi, 2018). More specifically in ESCs, genes have the active chromatin mark at their promoters and the repressive chromatin marks within the body of the gene, known as bivalency (Harikumar and Meshorer, 2015). These genes seem to fall into the category of genes that are required for future development of the embryo and differentiation. This bivalency was revealed using chromatin immunoprecipitation ChIP (Bernstein et al., 2006).

Although, the epigenome of any cell can be altered by the cell itself and by various drugs applied through the medium, it remains that ATP-chromatin modeling, histone modification and DNA methylation are critical in tightly regulating the journey of a stem cell, whether it be embryonic, an induced pluripotent or otherwise. Interestingly, a stem cell may have a different epigenetic code to its parent cell, allowing them to be flexible in becoming which ever lineage they are signaled to become. In iPSCs reprogramming with the transcription factors (*OCT4, SOX2, KLF4*, and *c-MYC*) leads to the resetting of the epigenome (Papp and Plath, 2011), with DNA demethylation leading to the active transcription of pluripotency genes (He et al., 2017). There is concern and evidence that there is an epigenetic memory in iPSCs that could remain in the genomes (Papp and Plath, 2011; Godini and Fallahi, 2018), with the possibility that this leads to instability later in their differentiation journeys. Indeed, in low methylated regions this epigenetic memory lasts for many passages. Whereas, in hypomethylated and hypermethylated genomic memories are located at conserved sites for active gene expression (Luu et al., 2018). With respect to DNA cytosine methylation in preimplantation embryos, DNA is hypomethylated, allowing for a poised/active gene state, with a global remethylation commencing at implantation (Guo et al., 2014; Okae et al., 2014). Indeed, DNA methylation is critical in cell fate, being directly involved in gene expression in pluripotency (Singer et al., 2014).

Studies have been performed to compare the epigenetic landscape of iPSCs with ESCs, to determine their similarity. Indeed, there are a number of differences (Bilic and Belmonte, 2012). These differences may be due to variations within populations since when ESCs and iPSCs were derived from the same origin there were no differences (Mallon et al., 2014). Thus, it could be argued that to be of clinical use iPSCs should be screened for specific histone marks and DNA methylation status of a selected panel of genes prior to being used.

## Current Methods for Aneuploidy Detection

Preimplantation genetic screening is commonly performed on human IVF embryos for an increased likelihood of a healthy birth (Munné et al., 1995), as it has been estimated that over 70% of normally developing human preimplantation embryos have chromosomal abnormalities (van Echten-Arends et al., 2011; Mertzanidou et al., 2013). As previously mentioned, the effects of low-level of aneuploidies in hPSCs are unknown and pose a serious threat to their therapeutic use because of their growth advantage in culture and tumorigenic potential, therefore is vital that they are well-characterized before use. For hPSCs to become a future treatment option for patients, especially for cell and gene therapies with a short shelf life, fast and robust methods for the sensitive detection of chromosomal abnormalities must be used. Currently, a number of different methods are available for such screening, each with their advantages and disadvantages with regards to sensitivity, resolution, turnover time, cost and staff requirement. Commonly used assays to detect chromosomal abnormalities are listed in Table 2.

**Table 2:** Current methods used for aneuploidy detection and their individual sensitivities.

**Method**	**Sensitivity of aneuploidy detection**
qPCR	10% (D'Hulst et al., 2013)
G-Banding	5–10% (Baker et al., 2007)
FISH	1–5% (Downie et al., 1997; Baker et al., 2007)
CGH	10–25% (Lu et al., 2007; Xiang et al., 2008; Manning et al., 2010; Novik et al., 2014)
dPCR	≤5% (El Khattabi et al., 2016)
NGS	<1% (Sachdeva et al., 2017)

The most common method utilized for aneuploidy detection is G-banding of metaphase chromosome spreads. The traditional technique that uses a dye to stain and observe specific banding patterns in condensed chromosomes is highly labor-intensive and requires trained cytogenetics for analysis. Typically, only 10–30 metaphase spreads are analyzed to assign a karyotype for the whole population, limiting the sensitivity of such a method. In addition, this creates difficulty in the detection of low-level mosaicism in culture; it has been estimated that only up to 5–10% mosaic aneuploidy detection is possible using G-banding (Baker et al., 2007) (Table 1), and can often fail to observe genomic imbalances less than 10 Mbs (Miller et al., 2010). In addition, G-banding results can be interpreted differently by different cytogeneticists resulting in inconsistent outcomes; it is also known for high turnaround times. Despite these drawbacks, G-banding results in a single-cell analysis, and examines every chromosome in each cell analyzed, unlike other cytogenetic-based methods, can detect balanced translocations and is relatively cost-efficient.

Another common cytogenetic method, fluorescence *in-situ* hybridization (FISH), employing a DNA specific probe in metaphase or interphase cells, also works at the single-cell level, with the option of multiplexing using different colored labels for each chromosome (mFISH), which can greatly aid the interpretation of complex translocations. FISH has been estimated to be approximately 1 to 5% sensitive for the detection of specific aneuploidies (Downie et al., 1997; Baker et al., 2007), can be carried out and analyzed within a few days and is once again, relatively cost-effective. However, the technique is still labor-intensive and requires the use of targeted probes for known abnormalities. FISH, could be successfully utilized as a sensitive screening method before the therapeutic use of hPSCs if designed for common aneuploid chromosomes.

The technique of multiplex ligation-dependent probe amplification (MLPA), originally designed to measure gene-copy number variations, can also be applied to detect aneuploidies of specific chromosomes. MLPA is designed to work by the detection of gene dosage abnormalities by utilizing up to 45 different DNA sequences. Rather than amplify the nucleic acids in the sample, the technique amplifies the probes that are added to the sample; the amplification depends on the presence of specific sequences in the sample. The probe intensities are quantified and the whole experiment typically takes 2–3 days (Sellner and Taylor, 2004; Shaffer, 2007). While MLPA is high-throughput and cost-effective, its sensitivity for detection of mosaicism is unclear, but likely does not exceed 10% (van Veghel-Plandsoen et al., 2011; Yan et al., 2011) and cannot detect structural aberrations.

Quantitative PCR (QPCR) may also be employed for the measurement of gains and losses of specific sequences; multiple short tandem repeats of the common aneuploidies in live births, such as trisomy 13, 18, and 21 and the sex chromosome aneuploidies can be used to amplify the regions of interest. The method allows the multiplexing of different fluorescence intensities produced from the PCR, resulting in a fast method for chromosome copy number detection. Studies have demonstrated 99.2% accuracy for whole chromosome aneuploidy detection in prenatal diagnosis (Cirigliano et al., 2004; Ogilvie et al., 2005), however, a level of sensitivity of 10% has been claimed for aneuploidy detection in mESCs (D'Hulst et al., 2013). Furthermore, QPCR has been demonstrated to be able to detect the presence of 20–30% mosaicism (Donaghue et al., 2005) and has been reported to be a much cheaper and faster alternative to other assays and many laboratories have now replaced traditional FISH with QF-PCR (Shaffer, 2007). Unfortunately, the limitation of QF-PCR is the inability to detect balanced chromosomal translocations and the assay's sensitivity is limited by the measurement of cycle-threshold differences. Digital PCR (dPCR), a more novel and sensitive technique, employs the same chemistry and amplification process as QPCR, therefore its potential for detecting mosaicism and future potential is much greater (Uchiyama et al., 2016). In the massively parallel partitioning of single-target molecule PCR reactions, dPCR has much greater power than QPCR to detect subtle difference in copy-number. In an analysis of trisomy 21 DNA samples, mosaicism for chromosome 21 was detected as low as 5% sensitivity (El Khattabi et al., 2016). With a greater number of replicate samples and a battery of assays for each chromosome arm, it is feasible that dPCR could be capable of detecting genome-wide aneuploidy to a level close to 1%.

Alternatively, chromosomal microarray methods, such as array-based comparative genomic hybridization (aCGH), KaryoLite-Bac on Beads^®^ (KaryoLite BoBs^®^) and single-nucleotide polymorphism techniques can also be used to detect aneuploidies. DNA microarrays use a panel of DNA sequences that compare the copy number of each area of interest to a control to then calculate the gene copy number of the sample (Shaffer, 2007). The advantage of using a DNA microarray is the ability to construct the target molecules, although most commonly the pre-designed, commercially-available microarray platforms for aCGH are used. The technique can detect DNA dosage imbalances, such as aneuploidies, deletions and duplications with a high resolution, dependent on the target of interest and is a much faster approach than methods, such as FISH, due to the high-throughput data produced. In addition, these techniques provide a significantly higher resolution of up to 50–500 kbs (Coe et al., 2007; Askree et al., 2013; WiCell, 2017), but in contrast to FISH and G-banding, the detection sensitivity of mosaicism is only about 10–25% (Lu et al., 2007; Xiang et al., 2008; Manning et al., 2010; Novik et al., 2014) but has been reported to be capable of detecting aneuploidy mosaicism as low as 5% (Menten et al., 2006), although such high levels of sensitivity are uncommon.

The evolution of next-generation sequencing (NGS) based methodologies extends the possible breadth of data which may be collected on molecular-level changes including at the single cell level. Whole genome sequencing may allow capture of the entire DNA sequence, whilst whole exome sequencing may offer a more affordable approach; both are challenged by some sequence variables including mononucleotide repeats, translocations, inversions, and large copy number variations. Targeted-panels, particularly for cancer-associated variants (such as those routinely used in cancer diagnostics) may provide focused data on known-impact genomic changes and also enable, through a higher number of reads per base pair sequenced, the detection of sub-clonal mutations down to a level of ~10% of cells. In a study analysing cells from hundreds of pre-implantation embryos with whole genome NGS very high sensitivity and specificity for aneuploidy of all chromosomes was reached (Sachdev et al., 2017), which could be described as a detection sensitivity of < 1%. NGS is also useful to assess the genomic health of PSCs by being employed in RNA-seq and ChIP-seq (Kidder et al., 2011; Zhang et al., 2013). Interestingly, RNA-Seq of PSCs with additional chromosomes reveals that transcription is affected across the whole genome, even for chromosomes and genes that have a normal copy number (Zhang et al., 2013). This consequence of aneuploidy is potentially dramatic if these cells survive in a body.

Additionally, newer karyotyping methods have been developed to use the changes in global gene expression changes to monitor chromosomal aberrations (Mayshar et al., 2010; Weissbein et al., 2016). Such methods could be used be in the future to determine the cell karyotype, however further work is required to detect the method's sensitivity in detecting chromosomal abnormalities. In addition, testing of different cell culture conditions would be required, as changes in gene expression would be detected with changes in the stem cell growth condition.

A challenge lies, even in the advent of highly sensitive aneuploidy-detection methods, in determining what confers an unacceptable level of genomic instability in hPSCs. Much data may be collected on genomic alterations in *in vitro* studies, but until there is a consensus on what safe limits may be, there is a risk of being overly cautious or hasty in realizing their therapeutic potential.

## Conclusion

Chromosomal aneuploidies in hPSCs can impair differentiation potential (Zhang et al., 2016) and potentially lead to tumorgenicity (Blum and Benvenisty, 2008; Ben-David and Benvenisty, 2011), which could limit their future therapeutic use. Studies on the genomic instability of hPSCs in culture are ongoing to optimize protocols for best practice. However, the ability of aneuploid cells to revert to diploid status over time in culture should not be overlooked, as observed with trisomy 18 hiPSCs (Li et al., 2017). Furthermore, some studies have demonstrated that an aberrant karyotype may not affect the quality of human preimplantation embryos (Mertzanidou et al., 2013), and indeed using mosaic embryos may still result in newborns with a normal karyotype (Greco et al., 2015). Although these studies are encouraging for the employment of embryos for preimplantation, their use must still be questionable, due to the possibility of future malignancy (Amariglio et al., 2009) and findings may not be transferable to using hPSCs in a similar state.

The high rate of aneuploidies observed in PSCs arises from a number of possible mechanisms and we have highlighted impaired mechanisms that affect mitotic segregation of chromosomes such as DNA damage, lamin B depletion, DNA damage repair, spindle assembly and checkpoint function. There are also important differences in the way the genome is organized and interacted with in interphase nuclei. The epigenome is also significantly different between PSCs and differentiated cells, seeming much more “malleable” prior to differentiation. The impact of aneuploidy on the epigenome is not clear and needs further exploration.

The prevalence of aneuploidies in PSCs in culture appears to be driven by the selection of genes which promote survival during periods of cell stress or offer a growth advantage. To move forward in the use of embryonic or induced pluripotent stem cells as therapeutics, methods that can easily be established in the clinic need should be considered for the high-throughput and sensitive detection of aneuploidies, such as population and single cell NGS, Hi-C, ChIP-seq, and RNA-Seq. However, much more research is required to determine any long-term detrimental effects using heterogenous stem cell cultures with respect to genomic content and behavior traits, nuclear architecture and content, and the epigenome. This will create the knowledge for the field to agree what constitutes a safe, acceptable limit of genomic instability in pluripotent cells.

## Author Contributions

JB and JMB are both corresponding authors, added to the review and oversaw the completion of the manuscript. MH has done most of the writing as primary author. JH wrote parts of the review and also was involved in the final versions of the manuscript.

### Conflict of Interest Statement

The authors declare that the research was conducted in the absence of any commercial or financial relationships that could be construed as a potential conflict of interest.

## References

[B1] Adida C. Crotty P. L. McGrath J. Berrebi D. Diebold J. Altieri D. C. (1998). Developmentally regulated expression of the novel cancer anti-apoptosis gene survivin in human and mouse differentiation. Am. J. Pathol. 152, 43–49. 9422522 PMC1858132

[B2] Aebi U. Cohn J. Buhle L. Gerace L. (1986). The nuclear lamina is a meshwork of intermediate-type filaments. Nature 323, 560–564. 10.1038/323560a0 3762708

[B3] Alcobia I. Dilão R. Parreira L. (2000). Spatial associations of centromeres in the nuclei of hematopoietic cells: evidence for cell-type-specific organizational patterns. Blood 95, 1608–1615. 10688815

[B4] Amariglio N. Hirshberg A. Scheithauer B. W. Cohen Y. Loewenthal R. Trakhtenbrot L.. (2009). Donor-derived brain tumor following neural stem cell transplantation in an ataxia telangiectasia patient. PLoS Med. 6:e1000029. 10.1371/journal.pmed.1000029 19226183 PMC2642879

[B5] Amendola M. van Steensel B. (2014). Mechanisms and dynamics of nuclear lamina–genome interactions. Curr. Opin. Cell Biol. 28, 61–68. 10.1016/j.ceb.2014.03.003 24694724

[B6] Amps K. Andrews P. W. Anyfantis G. Armstrong L. Avery S. Baharvand H.. (2011). Screening ethnically diverse human embryonic stem cells identifies a chromosome 20 minimal amplicon conferring growth advantage. Nat. Biotechnol. 29, 1132–1144. 10.1038/nbt.2051 22119741 PMC3454460

[B7] Andoh T. Ishida R. (1998). Catalytic inhibitors of DNA topoisomerase II. Biochim. Biophys. Acta 1400, 155–171. 10.1016/S0167-4781(98)00133-X 9748552

[B8] Apostolou E. Ferrari F. Walsh R. M. Bar-Nur O. Stadtfeld M. Cheloufi S.. (2013). Genome-wide chromatin interactions of the Nanog locus in pluripotency, differentiation, and reprogramming. Cell Stem Cell 12, 699–712. 10.1016/j.stem.2013.04.013 23665121 PMC3725985

[B9] Armstrong L. Saretzki G. Peters H. Wappler I. Evans J. Hole N.. (2005). Overexpression of telomerase confers growth advantage, stress resistance, and enhanced differentiation of ESCs toward the hematopoietic lineage. Stem Cells 23, 516–529. 10.1634/stemcells.2004-0269 15790773

[B10] Artus J. Babinet C. Cohen-Tannoudji M. (2006). The cell cycle of early mammalian embryos: lessons from genetic mouse models. Cell Cycle 5, 499–502. 10.4161/cc.5.5.2500 16481745

[B11] Askree S. H. Chin E. L. H. Bean L. H. Coffee B. Tanner A. Hegde M. (2013). Detection limit of intragenic deletions with targeted array comparative genomic hybridization. BMC Genet. 14:116. 10.1186/1471-2156-14-116 24304607 PMC4235222

[B12] Avery S. Hirst A. J. Baker D. Lim C. Y. Alagaratnam S. Skotheim R. I.. (2013). BCL-XL mediates the strong selective advantage of a 20q11.21 amplification commonly found in human embryonic stem cell cultures. Stem Cell Rep. 1, 379–386. 10.1016/j.stemcr.2013.10.005 24286026 PMC3841249

[B13] Azzam E. I. de Toledo S. M. Gooding T. Little J. B. (1998). Intercellular communication is involved in the bystander regulation of gene expression in human cells exposed to very low fluences of alpha particles. Radiat. Res. 150, 497–504. 9806590

[B14] Baarlink C. Wang H. Grosse R. (2013). Nuclear actin network assembly by formins regulates the SRF coactivator MAL. Science 340, 864–867. 10.1126/science.1235038 23558171

[B15] Badylak S. F. Taylor D. Uygun K. (2011). Whole-organ tissue engineering: decellularization and recellularization of three-dimensional matrix scaffolds. Annu. Rev. Biomed. Eng. 13, 27–53. 10.1146/annurev-bioeng-071910-124743 21417722 PMC10887492

[B16] Baker D. E. Harrison N. J. Maltby E. Smith K. Moore H. D. Shaw P. J.. (2007). Adaptation to culture of human embryonic stem cells and oncogenesis *in vivo*. Nat. Biotechnol. 25, 207–215. 10.1038/nbt1285 17287758

[B17] Baker M. (2009). Unregulated stem cell transplant causes tumours. Nat. Rep. Stem Cells. 10.1038/stemcells.2009.32

[B18] Balasubramanian B. Pogozelski W. K. Tullius T. D. (1998). DNA strand breaking by the hydroxyl radical is governed by the accessible surface areas of the hydrogen atoms of the DNA backbone. Chemistry 95, 9738–9743. 9707545 10.1073/pnas.95.17.9738 PMC21406

[B19] Barker N. van Es J. H. Kuipers J. Kujala P. van den Born M. Cozijnsen M.. (2007). Identification of stem cells in small intestine and colon by marker gene Lgr5. Nature 449, 1003–1007. 10.1038/nature06196 17934449

[B20] Barker R. A. Parmar M. Kirkeby A. Björklund A. Thompson L. Brundin P. (2016). Are stem cell-based therapies for parkinson's disease ready for the clinic in 2016? J. Parkinsons Dis. 6, 57–63. 10.3233/JPD-160798 27003785 PMC4927930

[B21] Bártová E. Galiová G. Krejcí J. Harnicarová A. Strašák L. Kozubek S.. (2008). Epigenome and chromatin structure in human embryonic stem cells undergoing differentiation. Dev. Dyn. 237, 3690–3702. 10.1002/dvdy.21773 18985715

[B22] Bártová E. Kozubek S. Jirsová P. Kozubek M. Lukásová E. Skalníková M.. (2001). Higher-order chromatin structure of human granulocytes. Chromosoma 110, 360–370. 10.1007/s004120100141 11685536

[B23] Baumann C. Körner R. Hofmann K. Nigg E. A. (2007). PICH, a centromere-associated SNF2 family ATPase, is regulated by Plk1 and required for the spindle checkpoint. Cell 128, 101–114. 10.1016/j.cell.2006.11.041 17218258

[B24] Becker K. A. Ghule P. N. Therrien J. A. Lian J. B. Stein J. L. van Wijnen A. J.. (2006). Self-renewal of human embryonic stem cells is supported by a shortened G1 cell cycle phase. J. Cell. Physiol. 209, 883–893. 10.1002/jcp.20776 16972248

[B25] Beil M. Dürschmied D. Paschke S. Schreiner B. Nolte U. Bruel A.. (2002). Spatial distribution patterns of interphase centromeres during retinoic acid-induced differentiation of promyelocytic leukemia cells. Cytometry 47, 217–225. 10.1002/cyto.10077 11933011

[B26] Ben-David U. Arad G. Weissbein U. Mandefro B. Maimon A. Golan-Lev T.. (2014). Aneuploidy induces profound changes in gene expression, proliferation and tumorigenicity of human pluripotent stem cells. Nat. Commun. 5:4825. 10.1038/ncomms5825 25198699

[B27] Ben-David U. Benvenisty N. (2011). The tumorigenicity of human embryonic and induced pluripotent stem cells. Nat. Rev. Cancer 11, 268–277. 10.1038/nrc3034 21390058

[B28] Bernstein B. E. Mikkelsen T. S. Xie X. Kamal M. Huebert D. J. Cuff J.. (2006). A bivalent chromatin structure marks key developmental genes in embryonic stem cells. Cell 125, 315–326. 10.1016/j.cell.2006.02.041 16630819

[B29] Bickmore W. A. van Steensel B. (2013). Genome architecture: domain organization of interphase chromosomes. Cell 152, 1270–1284. 10.1016/j.cell.2013.02.001 23498936

[B30] Bilic J. Belmonte J. C. (2012). Concise review: induced pluripotent stem cells versus embryonic stem cells: close enough or yet too far apart? Stem Cells 30, 33–41. 10.1002/stem.700 22213481

[B31] Bjorklund L. M. Sánchez-Pernaute R. Chung S. Andersson T. Chen I. Y. C. McNaught K. S. P.. (2002). Embryonic stem cells develop into functional dopaminergic neurons after transplantation in a Parkinson rat model. Proc. Natl. Acad. Sci. U.S.A. 99, 2344–2349. 10.1073/pnas.022438099 11782534 PMC122367

[B32] Blum B. Benvenisty N. (2008). The tumorigenicity of human embryonic stem cells. Adv. Cancer Res. 100, 133–158. 10.1016/S0065-230X(08)00005-5 18620095

[B33] Boise L. H. González-García M. Postema C. E. Ding L. Lindsten T. Turka L. A.. (1993). bcl-x, a bcl-2-related gene that functions as a dominant regulator of apoptotic cell death. Cell 74, 597–608. 8358789 10.1016/0092-8674(93)90508-n

[B34] Bonnet-Garnier A. Kiêu K. Aguirre-Lavin T. Tar K. Flores P. Liu Z.. (2018). Three-dimensional analysis of nuclear heterochromatin distribution during early development in the rabbit. Chromosoma 127, 387–403. 10.1007/s00412-018-0671-z 29666907 PMC6096579

[B35] Boyle S. Gilchrist S. Bridger J. M. Mahy N. L. Ellis J. A. Bickmore W. A. (2001). The spatial organization of human chromosomes within the nuclei of normal and emerin-mutant cells. Hum. Mol. Genet. 10, 211–219. 10.1093/hmg/10.3.211 11159939

[B36] Briand N. Cahyani I. Madsen-Østerbye J. Paulsen J. Rønningen T. Sørensen A. L.. (2018). Lamin A, chromatin and FPLD2: not just a peripheral ménage-à-trois. Front. Cell. Dev. Biol. 6:73. 10.3389/fcell.2018.00073 30057899 PMC6053905

[B37] Bridger J. M. Arican-Gotkas H. D. Foster H. A. Godwin L. S. Harvey A. Kill I. R.. (2014). The non-random repositioning of whole chromosomes and individual gene loci in interphase nuclei and its relevance in disease, infection, aging, and cancer. Adv. Exp. Med. Biol. 773, 263–279. 10.1007/978-1-4899-8032-8_12 24563352

[B38] Bridger J. M. Bickmore W. A. (1998). Putting the genome on the map. Trends Genet. 14, 403–409. 10.1016/S0168-9525(98)01572-8 9820029

[B39] Bridger J. M. Boyle S. Kill I. R. Bickmore W. A. (2000). Re-modelling of nuclear architecture in quiescent and senescent human fibroblasts. Curr. Biol. 10, 149–152. 10.1016/S0960-9822(00)00312-2 10679329

[B40] Bridger J. M. Foeger N. Kill I. R. Herrmann H. (2007). The nuclear lamina. FEBS J. 274, 1354–1361. 10.1111/j.1742-4658.2007.05694.x 17489093

[B41] Bridger J. M. Mehta I. S. (2011). Nuclear molecular motors for active, directed chromatin movement in interphase nuclei, in Advances in Nuclear Architecture (Dordrecht: Springer), 149–172. 10.1007/978-90-481-9899-3_5

[B42] Brimble S. N. Zeng X. Weiler D. A. Luo Y. Liu Y. Lyons I. G.. (2004). Karyotypic stability, genotyping, differentiation, feeder-free maintenance, and gene expression sampling in three human embryonic stem cell lines derived prior to August 9, 2001. Stem Cells Dev. 13, 585–597. 10.1089/scd.2004.13.585 15684826

[B43] Brown A. Geiger H. (2018). Chromosome integrity checkpoints in stem and progenitor cells: transitions upon differentiation, pathogenesis, and aging. Cell. Mol. Life Sci. 75, 3771–3779. 10.1007/s00018-018-2891-z 30066086 PMC6154040

[B44] Bunch J. T. Bae N. S. Leonardi J. Baumann P. (2005). Distinct requirements for Pot1 in limiting telomere length and maintaining chromosome stability. Mol. Cell. Biol. 25, 5567–5578. 10.1128/MCB.25.13.5567-5578.2005 15964812 PMC1156986

[B45] Butler J. T. Hall L. L. Smith K. P. Lawrence J. B. (2009). Changing nuclear landscape and unique PML structures during early epigenetic transitions of human embryonic stem cells. J. Cell. Biochem. 107, 609–621. 10.1002/jcb.22183 19449340 PMC2937361

[B46] Cai M. (2001). Solution structure of the constant region of nuclear envelope protein LAP2 reveals two LEM-domain structures: one binds BAF and the other binds DNA. EMBO J. 20, 4399–4407. 10.1093/emboj/20.16.4399 11500367 PMC125263

[B47] Caine A. Maltby a,. E. Parkin C. A. Waters J. J. Crolla J. a. (2005). Prenatal detection of Down's syndrome by rapid aneuploidy testing for chromosomes 13, 18, and 21 by FISH or PCR without a full karyotype: a cytogenetic risk assessment. Lancet 366, 123–128. 10.1016/S0140-6736(05)66790-6 16005334

[B48] Card D. A. Hebbar P. B. Li L. Trotter K. W. Komatsu Y. Mishina Y.. (2008). Oct4/Sox2-regulated miR-302 targets cyclin D1 in human embryonic stem cells. Mol. Cell. Biol. 28, 6426–6438. 10.1128/MCB.00359-08 18710938 PMC2577422

[B49] Castellanos E. Dominguez P. Gonzalez C. (2008). Centrosome dysfunction in Drosophila neural stem cells causes tumors that are not due to genome instability. Curr. Biol. 18, 1209–1214. 10.1016/j.cub.2008.07.029 18656356

[B50] Caussinus E. Gonzalez C. (2005). Induction of tumor growth by altered stem-cell asymmetric division in *Drosophila melanogaster*. Nat. Genet. 37, 1125–1129. 10.1038/ng1632 16142234

[B51] Chan K.-L. North P. S. Hickson I. D. (2007). BLM is required for faithful chromosome segregation and its localization defines a class of ultrafine anaphase bridges. EMBO J. 26, 3397–3409. 10.1038/sj.emboj.7601777 17599064 PMC1933408

[B52] Chen K. G. Mallon B. S. McKay R. D. G. Robey P. G. (2014). Human pluripotent stem cell culture: considerations for maintenance, expansion, and therapeutics. Cell Stem Cell. 14, 13–26. 10.1016/j.stem.2013.12.005 24388173 PMC3915741

[B53] Chung M. K. Jeong H. J. Lee J. H. Park S.-J. Chung H.-D. Kang H.-Y. (2013). Comprehensive chromosome analysis of blastocysts before implantation using array CGH. Mol. Cytogenet. 6:22. 10.1186/1755-8166-6-22 23731833 PMC3720204

[B54] Cimini D. Fioravanti D. Salmon E. D. Degrassi F. (2002). Merotelic kinetochore orientation versus chromosome mono-orientation in the origin of lagging chromosomes in human primary cells. J. Cell Sci. 115, 507–515. 11861758 10.1242/jcs.115.3.507

[B55] Cimini D. Tanzarella C. Degrassi F. (1999). Differences in malsegregation rates obtained by scoring ana-telophases or binucleate cells. Mutagenesis 14, 563–568. 10567031 10.1093/mutage/14.6.563

[B56] Cirigliano V. Voglino G. Cañadas M. P. Marongiu A. Ejarque M. Ordoñez E.. (2004). Rapid prenatal diagnosis of common chromosome aneuploidies by QF-PCR. Assessment on 18 000 consecutive clinical samples. MHR Basic Sci. Reprod. Med. 10, 839–846. 10.1093/molehr/gah108 15361554

[B57] Clements C. S. Bikkul U. Ahmed M. H. Foster H. A. Godwin L. S. Bridger J. M. (2016). Visualizing the Spatial Relationship of the Genome with the Nuclear Envelope Using Fluorescence In Situ Hybridization. New York, NY: Humana Press, 387–406. 10.1007/978-1-4939-3530-7_24 27147055

[B58] Coe B. P. Ylstra B. Carvalho B. Meijer G. A. MacAulay C. Lam W. L. (2007). Resolving the resolution of array CGH. Genomics 89, 647–653. 10.1016/j.ygeno.2006.12.012 17276656

[B59] Coffinier C. Chang S. Y. Nobumori C. Tu Y. Farber E. A. Toth J. I.. (2010). Abnormal development of the cerebral cortex and cerebellum in the setting of lamin B2 deficiency. Proc. Natl. Acad. Sci. U.S.A. 107, 5076–5081. 10.1073/pnas.0908790107 20145110 PMC2841930

[B60] Constantinescu D. Gray H. L. Sammak P. J. Schatten G. P. Csoka A. B. (2006). Lamin A/C expression is a marker of mouse and human embryonic stem cell differentiation. Stem Cells 24, 177–185. 10.1634/stemcells.2004-0159 16179429

[B61] Cremer T. Cremer C. (2001). Chromosome territories, nuclear architecture and gene regulation in mammalian cells. Nat. Rev. Genet. 2, 292–301. 10.1038/35066075 11283701

[B62] Criscione S. W. Teo Y. V. Neretti N. (2016). The chromatin landscape of cellular senescence. Trends Genet. 32, 751–761. 10.1016/j.tig.2016.09.005 27692431 PMC5235059

[B63] Croft J. A. Bridger J. M. Boyle S. Perry P. Teague P. Bickmore W. A. (1999). Differences in the localization and morphology of chromosomes in the human nucleus. J. Cell Biol. 145, 1119–1131. 10.1083/jcb.145.6.1119 10366586 PMC2133153

[B64] Czapiewski R. Robson M. I. Schirmer E. C. (2016). Anchoring a leviathan: how the nuclear membrane tethers the genome. Front. Genet. 7:82. 10.3389/fgene.2016.00082 27200088 PMC4859327

[B65] Damelin M. Sun Y. E. Sodja V. B. Bestor T. H. (2005). Decatenation checkpoint deficiency in stem and progenitor cells. Cancer Cell 8, 479–484. 10.1016/j.ccr.2005.11.004 16338661

[B66] de Wit E. Bouwman B. A. M. Zhu Y. Klous P. Splinter E. Verstegen M. J. A. M.. (2013). The pluripotent genome in three dimensions is shaped around pluripotency factors. Nature 501, 227–231. 10.1038/nature12420 23883933

[B67] Dechat T. Pfleghaar K. Sengupta K. Shimi T. Shumaker D. K. Solimando L.. (2008). Nuclear lamins: major factors in the structural organization and function of the nucleus and chromatin. Genes Dev. 22, 832–853. 10.1101/gad.1652708 18381888 PMC2732390

[B68] DePinho R. A. (2000). The age of cancer. Nature 408, 248–254. 10.1038/35041694 11089982

[B69] Deshpande A. Goodwin E. H. Bailey S. M. Marrone B. L. Lehnert B. E. (1996). Alpha-particle-induced sister chromatid exchange in normal human lung fibroblasts: evidence for an extranuclear target. Radiat. Res. 145, 260–267. 8927692

[B70] Deshpande D. M. Kim Y.-S. Martinez T. Carmen J. Dike S. Shats I.. (2006). Recovery from paralysis in adult rats using embryonic stem cells. Ann. Neurol. 60, 32–44. 10.1002/ana.20901 16802299

[B71] Desmarais J. A. Hoffmann M. J. Bingham G. Gagou M. E. Meuth M. Andrews P. W. (2012). Human embryonic stem cells fail to activate CHK1 and commit to apoptosis in response to DNA replication stress. Stem Cells 30, 1385–1393. 10.1002/stem.1117 22553144

[B72] D'Hulst C. Parvanova I. Tomoiaga D. Sapar M. L. Feinstein P. (2013). Fast quantitative real-time pcr-based screening for common chromosomal aneuploidies in mouse embryonic stem cells. Stem Cell Rep. 1, 350–359. 10.1016/j.stemcr.2013.08.003 24319669 PMC3849352

[B73] Di Stefano B. Ueda M. Sabri S. Brumbaugh J. Huebner A. J. Sahakyan A.. (2018). Reduced MEK inhibition preserves genomic stability in naive human embryonic stem cells. Nat. Methods 15, 732–740. 10.1038/s41592-018-0104-1 30127506 PMC6127858

[B74] Ding X. Xu R. Yu J. Xu T. Zhuang Y. Han M. (2007). SUN1 is required for telomere attachment to nuclear envelope and gametogenesis in mice. Dev. Cell 12, 863–872. 10.1016/j.devcel.2007.03.018 17543860

[B75] Dixon J. R. Selvaraj S. Yue F. Kim A. Li Y. Shen Y.. (2012). Topological domains in mammalian genomes identified by analysis of chromatin interactions. Nature 485, 376–380. 10.1038/nature11082 22495300 PMC3356448

[B76] Dolezalova D. Mraz M. Barta T. Plevova K. Vinarsky V. Holubcova Z. (2012). MicroRNAs regulate p21Waf1/Cip1 Protein expression and the DNA damage response in human embryonic stem cells. Stem Cells 30, 1362–1372. 10.1002/stem.1108 22511267

[B77] Donaghue C. Mann K. Docherty Z. Mackie Ogilvie C. (2005). Detection of mosaicism for primary trisomies in prenatal samples by QF-PCR and karyotype analysis. Prenat. Diagn. 25, 65–72. 10.1002/pd.1086 15662691

[B78] Downie S. E. Flaherty S. P. Matthews C. D. (1997). Detection of chromosomes and estimation of aneuploidy in human spermatozoa using fluorescence *in-situ* hybridization. Mol. Hum. Reprod. 3, 585–598. 9268136 10.1093/molehr/3.7.585

[B79] Draper J. S. Smith K. Gokhale P. Moore H. D. Maltby E. Johnson J.. (2004). Recurrent gain of chromosomes 17q and 12 in cultured human embryonic stem cells. Nat. Biotechnol. 22, 53–54. 10.1038/nbt922 14661028

[B80] Dürrbaum M. Storchová Z. (2016). Effects of aneuploidy on gene expression: implications for cancer. FEBS J. 283, 791–802. 10.1111/febs.13591 26555863

[B81] Eckersley-Maslin M. A. Bergmann J. H. Lazar Z. Spector D. L. (2013). Lamin A/C is expressed in pluripotent mouse embryonic stem cells. Nucleus 4, 53–60. 10.4161/nucl.23384 23324457 PMC3585028

[B82] El Khattabi L. A. Rouillac-Le Sciellour C. Le Tessier D. Luscan A. Coustier A. Porcher R.. (2016). Could digital PCR Be an alternative as a non-invasive prenatal test for trisomy 21: a proof of concept study. PLoS ONE 11:e0155009. 10.1371/journal.pone.0155009 27167625 PMC4864235

[B83] Enver T. Soneji S. Joshi C. Brown J. Iborra F. Orntoft T.. (2005). Cellular differentiation hierarchies in normal and culture-adapted human embryonic stem cells. Hum. Mol. Genet. 14, 3129–3140. 10.1093/hmg/ddi345 16159889

[B84] Espada J. Varela I. Flores I. Ugalde A. P. Cadiñanos J. Pendás A. M.. (2008). Nuclear envelope defects cause stem cell dysfunction in premature-aging mice. J. Cell Biol. 181, 27–35. 10.1083/jcb.200801096 18378773 PMC2287278

[B85] Farooq T. Rehman K. Hameed A. Akash M. S. H. (2018). Stem cell therapy and type 1 diabetes mellitus: treatment strategies and future perspectives. Adv. Exp. Med. Biol. 10.1007/5584_2018_195. [Epub ahead of print] 29896720

[B86] Fedorova E. Zink D. (2008). Nuclear architecture and gene regulation. Biochim. Biophys. Acta Mol. Cell Res. 1783, 2174–2184. 10.1016/J.BBAMCR.2008.07.018 18718493

[B87] Felgentreff K. Du L. Weinacht K. G. Dobbs K. Bartish M. Giliani S.. (2014). Differential role of nonhomologous end joining factors in the generation, DNA damage response, and myeloid differentiation of human induced pluripotent stem cells. Proc. Natl. Acad. Sci. U.S.A. 111, 8889–8894. 10.1073/pnas.1323649111 24889605 PMC4066476

[B88] Feng J. Funk W. D. Wang S. S. Weinrich S. L. Avilion A. A. Chiu C. P.. (1995). The RNA component of human telomerase. Science 269, 1236–1241. 7544491 10.1126/science.7544491

[B89] Ferguson M. Ward D. C. Manueiidis L. (1992). Cell cycle dependent chromosomal movement in pre-mitotic human T-lymphocyte nuclei. Chromosoma 101, 557–565. 1521500 10.1007/BF00660315

[B90] Filion T. M. Qiao M. Ghule P. N. Mandeville M. van Wijnen A. J. Stein J. L.. (2009). Survival responses of human embryonic stem cells to DNA damage. J. Cell. Physiol. 220, 586–592. 10.1002/jcp.21735 19373864 PMC2925401

[B91] Finch K. A. Fonseka G. Ioannou D. Hickson N. Barclay Z. Chatzimeletiou K.. (2008). Nuclear organisation in totipotent human nuclei and its relationship to chromosomal abnormality. J. Cell Sci. 121, 655–663. 10.1242/jcs.025205 18270263

[B92] Finkel T. Holbrook N. J. (2000). Oxidants, oxidative stress and the biology of ageing. Nature 408, 239–247. 10.1038/35041687 11089981

[B93] Flores I. Canela A. Vera E. Tejera A. Cotsarelis G. Blasco M. A. (2008). The longest telomeres: a general signature of adult stem cell compartments. Genes Dev. 22, 654–667. 10.1101/gad.451008 18283121 PMC2259034

[B94] Foster H. A. Abeydeera L. R. Griffin D. K. Bridger J. M. (2005). Non-random chromosome positioning in mammalian sperm nuclei, with migration of the sex chromosomes during late spermatogenesis. J. Cell Sci. 118, 1811–1820. 10.1242/jcs.02301 15827089

[B95] Foster H. A. Griffin D. K. Bridger J. M. (2012). Interphase chromosome positioning in *in vitro* porcine cells and *ex vivo* porcine tissues. BMC Cell Biol. 13:30. 10.1186/1471-2121-13-30 23151271 PMC3499214

[B96] Francastel C. Schübeler D. Martin D. I. Groudine M. (2000). Nuclear compartmentalization and gene activity. Nat. Rev. Mol. Cell Biol. 1, 137–143. 10.1038/35040083 11253366

[B97] Fritz B. Hallermann C. Olert J. Fuchs B. Bruns M. Aslan M.. (2001). Cytogenetic analyses of culture failures by comparative genomic hybridisation (CGH)-Re-evaluation of chromosome aberration rates in early spontaneous abortions. Eur. J. Hum. Genet. 9, 539–547. 10.1038/sj.ejhg.5200669 11464246

[B98] Galiová G. Bártová E. Kozubek S. (2004). Nuclear topography of β-like globin gene cluster in IL-3-stimulated human leukemic K-562 cells. Blood Cells Mol. Dis. 33, 4–14. 10.1016/j.bcmd.2004.03.006 15223004

[B99] Geiszt M. Kopp J. B. Várnai P. Leto T. L. (2000). Identification of renox, an NAD(P)H oxidase in kidney. Proc. Natl. Acad. Sci. U.S.A. 97, 8010–8014. 10.1073/pnas.130135897 10869423 PMC16661

[B100] Ghule P. N. Dominski Z. Yang X.-C. Marzluff W. F. Becker K. A. Harper J. W.. (2008). Staged assembly of histone gene expression machinery at subnuclear foci in the abbreviated cell cycle of human embryonic stem cells. Proc. Natl. Acad. Sci. U.S.A. 105, 16964–16969. 10.1073/pnas.0809273105 18957539 PMC2579361

[B101] Gilchrist S. Gilbert N. Perry P. Bickmore W. A. (2004). *Nuclear organization of centromeric domains is not perturbed by inhibition of histone* deacetylases. Chromosome Res. 12, 505–516. 10.1023/B:CHRO.0000034892.64739.ff 15254368

[B102] Gisselsson D. Jonson T. Petersén A. Strömbeck B. Dal Cin P. Höglund M.. (2001). Telomere dysfunction triggers extensive DNA fragmentation and evolution of complex chromosome abnormalities in human malignant tumors. Proc. Natl. Acad. Sci. U.S.A. 98, 12683–12688. 10.1073/pnas.211357798 11675499 PMC60114

[B103] Glinsky G. Durruthy-Durruthy J. Wossidlo M. Grow E. J. Weirather J. L. Au K. F.. (2018). Single cell expression analysis of primate-specific retroviruses-derived HPAT lincRNAs in viable human blastocysts identifies embryonic cells co-expressing genetic markers of multiple lineages. Heliyon 4:e00667. 10.1016/j.heliyon.2018.e00667 30003161 PMC6039856

[B104] Godini R. Fallahi H. (2018). Dynamics changes in the transcription factors during early human embryonic development. J. Cell. Physiol. [Epub ahead of print] 10.1002/jcp.27386 30246428

[B105] Goodarzi A. A. Noon A. T. Deckbar D. Ziv Y. Shiloh Y. Löbrich M.. (2008). ATM signaling facilitates repair of DNA double-strand breaks associated with heterochromatin. Mol. Cell 31, 167–177. 10.1016/j.molcel.2008.05.017 18657500

[B106] Gorbsky G. J. (1994). Cell cycle progression and chromosome segregation in mammalian cells cultured in the presence of the topoisomerase II inhibitors ICRF-187 [(+)-1,2-bis(3,5-dioxopiperazinyl-1-yl)propane; ADR-529] and ICRF-159 (Razoxane). Cancer Res. 54, 1042–1048. 8313360

[B107] Gotzmann J. Foisner R. (2006). A-type lamin complexes and regenerative potential: a step towards understanding laminopathic diseases? Histochem. Cell Biol. 125, 33–41. 10.1007/s00418-005-0050-8 16142451

[B108] Grealish S. Diguet E. Kirkeby A. Mattsson B. Heuer A. Bramoulle Y.. (2014). Human ESC-derived dopamine neurons show similar preclinical efficacy and potency to fetal neurons when grafted in a rat model of parkinson's disease. Cell Stem Cell 15, 653–665. 10.1016/j.stem.2014.09.017 25517469 PMC4232736

[B109] Greco E. Minasi M. G. Fiorentino F. (2015). Healthy babies after intrauterine transfer of mosaic aneuploid blastocysts. N. Engl. J. Med. 373, 2089–2090. 10.1056/NEJMc1500421 26581010

[B110] Greider C. W. Blackburn E. H. (1989). A telomeric sequence in the RNA of Tetrahymena telomerase required for telomere repeat synthesis. Nature 337, 331–337. 10.1038/337331a0 2463488

[B111] Gruenbaum Y. Wilson K. L. Harel A. Goldberg M. Cohen M. (2000). Review: nuclear lamins—structural proteins with fundamental functions. J. Struct. Biol. 129, 313–323. 10.1006/jsbi.2000.4216 10806082

[B112] Guenther M. G. Frampton G. M. Soldner F. Hockemeyer D. Mitalipova M. Jaenisch R.. (2010). Chromatin structure and gene expression programs of human embryonic and induced pluripotent stem cells. Cell Stem Cell 7, 249–257. 10.1016/j.stem.2010.06.015 20682450 PMC3010384

[B113] Guo F. Li X. Liang D. Li T. Zhu P. Guo H.. (2014). Active and passive demethylation of male and female pronuclear dna in the mammalian zygote. Cell Stem Cell 15, 447–459. 10.1016/j.stem.2014.08.003 25220291

[B114] Haaf T. Schmid M. (1988). Analysis of double minutes and double minute-like chromatin in human and murine tumor cells using antikinetochore antibodies. Cancer Genet. Cytogenet. 30, 73–82. 3275490 10.1016/0165-4608(88)90094-5

[B115] Hackett J. A. Feldser D. M. Greider C. W. (2001). Telomere dysfunction increases mutation rate and genomic instability. Cell 106, 275–286. 10.1016/S0092-8674(01)00457-3 11509177

[B116] Hadjimichael C. Chanoumidou K. Nikolaou C. Klonizakis A. Theodosi G.-I. Makatounakis T.. (2017). Promyelocytic leukemia protein is an essential regulator of stem cell pluripotency and somatic cell reprogramming. Stem Cell Rep. 8, 1366–1378. 10.1016/j.stemcr.2017.03.006 28392218 PMC5425614

[B117] Halaschek-Wiener J. Brooks-Wilson A. (2007). Progeria of stem cells: stem cell exhaustion in Hutchinson-Gilford progeria syndrome. J. Gerontol. A. Biol. Sci. Med. Sci. 62, 3–8. 10.1093/gerona/62.1.3 17301031

[B118] Harikumar A. Meshorer E. (2015). Chromatin remodeling and bivalent histone modifications in embryonic stem cells. EMBO Rep. 16, 1609–1619. 10.15252/embr.201541011 26553936 PMC4693513

[B119] Harrison N. J. Baker D. Andrews P. W. (2007). Culture adaptation of embryonic stem cells echoes germ cell malignancy. Int. J. Androl. 30, 275–281; discussion 281. 10.1111/j.1365-2605.2007.00762.x 17488340

[B120] He S. Sun H. Lin L. Zhang Y. Chen J. Liang L.. (2017). Passive DNA demethylation preferentially up-regulates pluripotency-related genes and facilitates the generation of induced pluripotent stem cells. J. Biol. Chem. 292, 18542–18555. 10.1074/jbc.M117.810457 28924038 PMC5682964

[B121] Hérault A. Binnewies M. Leong S. Calero-Nieto F. J. Zhang S. Y. Kang Y.-A.. (2017). Myeloid progenitor cluster formation drives emergency and leukaemic myelopoiesis. Nature 544, 53–58. 10.1038/nature21693 28355185 PMC5383507

[B122] Herberts C. A. Kwa M. S. G. Hermsen H. P. H. (2011). Risk factors in the development of stem cell therapy. J. Transl. Med. 9:29. 10.1186/1479-5876-9-29 21418664 PMC3070641

[B123] Hernandez L. Roux K. J. Wong E. S. M. Mounkes L. C. Mutalif R. Navasankari R.. (2010). Functional coupling between the extracellular matrix and nuclear lamina by wnt signaling in progeria. Dev. Cell 19, 413–425. 10.1016/j.devcel.2010.08.013 20833363 PMC2953243

[B124] Herszfeld D. Wolvetang E. Langton-Bunker E. Chung T.-L. Filipczyk A. A. Houssami S.. (2006). CD30 is a survival factor and a biomarker for transformed human pluripotent stem cells. Nat. Biotechnol. 24, 351–357. 10.1038/nbt1197 16501577

[B125] Hickman A. W. Jaramillo R. J. Lechner J. F. Johnson N. F. (1994). Alpha-particle-induced p53 protein expression in a rat lung epithelial cell strain. Cancer Res. 54, 5797–5800. 7954402

[B126] Hoffelder D. Luo L. Burke N. Watkins S. Gollin S. Saunders W. (2004). Resolution of anaphase bridges in cancer cells. Chromosoma 112, 389–397. 10.1007/s00412-004-0284-6 15156327

[B127] Höger T. H. Zatloukal K. Waizenegger I. Krohne G. (1990). Characterization of a second highly conserved B-type lamin present in cells previously thought to contain only a single B-type lamin. Chromosoma 99, 379–390. 2102682 10.1007/BF01726689

[B128] Holm C. Stearns T. Botstein D. (1989). DNA topoisomerase II must act at mitosis to prevent nondisjunction and chromosome breakage. Mol. Cell. Biol. 9, 159–168. 2538717 10.1128/mcb.9.1.159-168.1989 PMC362157

[B129] Horáková A. H. Bártová E. Kozubek S. (2010). Chromatin structure with respect to histone signature changes during cell differentiation. Cell Struct. Funct. 35, 31–44. 10.1247/csf.09021 20424340

[B130] Howman E. V. Fowler K. J. Newson A. J. Redward S. MacDonald A. C. Kalitsis P.. (2000). Early disruption of centromeric chromatin organization in centromere protein A (Cenpa) null mice. Proc. Natl. Acad. Sci. U.S.A. 97, 1148–1153. 10.1073/pnas.97.3.1148 10655499 PMC15551

[B131] Hu M. Krause D. Greaves M. Sharkis S. Dexter M. Heyworth C.. (1997). Multilineage gene expression precedes commitment in the hemopoietic system. Genes Dev. 11, 774–785. 9087431 10.1101/gad.11.6.774

[B132] Huang J. Wang F. Okuka M. Liu N. Ji G. Ye X.. (2011). Association of telomere length with authentic pluripotency of ES/iPS cells. Cell Res. 21, 779–792. 10.1038/cr.2011.16 21283131 PMC3203670

[B133] Hulspas R. Houtsmuller A. B. Krijtenburg P.-J. Bauman J. G. J. Nanninga N. (1994). The nuclear position of pericentromeric DNA of chromosome 11 appears to be random in G O and non-random in G 1 human lymphocytes. Chromosoma 103, 286–292. 7988290 10.1007/BF00352253

[B134] Hutchison C. J. (2002). Lamins: building blocks or regulators of gene expression? Nat. Rev. Mol. Cell Biol. 3, 848–858. 10.1038/nrm950 12415302

[B135] Huycke M. M. Abrams V. Moore D. R. (2002). Enterococcus faecalis produces extracellular superoxide and hydrogen peroxide that damages colonic epithelial cell DNA. Carcinogenesis 23, 529–536. 10.1093/carcin/23.3.529 11895869

[B136] Huycke M. M. Moore D. Joyce W. Wise P. Shepard L. Kotake Y.. (2001). Extracellular superoxide production by *Enterococcus faecalis* requires demethylmenaquinone and is attenuated by functional terminal quinol oxidases. Mol. Microbiol. 42, 729–740. 10.1046/j.1365-2958.2001.02638.x 11722738

[B137] Itoh N. Shimizu N. (1998). DNA replication-dependent intranuclear relocation of double minute chromatin. J. Cell Sci. 111 (Pt 2), 3275–3285. 9788870 10.1242/jcs.111.22.3275

[B138] Jiménez G. Griffiths S. D. Ford A. M. Greaves M. F. Enver T. (1992). Activation of the beta-globin locus control region precedes commitment to the erythroid lineage. Proc. Natl. Acad. Sci. U.S.A. 89, 10618–10622. 1438257 10.1073/pnas.89.22.10618 PMC50392

[B139] Kanemura H. Go M. J. Shikamura M. Nishishita N. Sakai N. Kamao H.. (2014). Tumorigenicity studies of induced pluripotent stem cell (iPSC)-derived retinal pigment epithelium (RPE) for the treatment of age-related macular degeneration. PLoS ONE 9:e85336. 10.1371/journal.pone.0085336 24454843 PMC3891869

[B140] Kapinas K. Kim H. Mandeville M. Martin-Buley L. A. Croce C. M. Lian J. B.. (2015). microRNA-mediated survivin control of pluripotency. J. Cell. Physiol. 230, 63–70. 10.1002/jcp.24681 24891298 PMC4182148

[B141] Kawamura T. Suzuki J. Wang Y. V. Menendez S. Morera L. B. Raya A.. (2009). Linking the p53 tumour suppressor pathway to somatic cell reprogramming. Nature 460, 1140–1144. 10.1038/nature08311 19668186 PMC2735889

[B142] Keohane A. M. O'neill L. P. Belyaev N. D. Lavender J. S. Turner B. M. (1996). X-Inactivation and histone H4 acetylation in embryonic stem cells. Dev. Biol. 180, 618–630. 8954732 10.1006/dbio.1996.0333

[B143] Kidder B. L. Hu G. Zhao K. (2011). ChIP-Seq: technical considerations for obtaining high-quality data. Nat. Immunol. 12, 918–922. 10.1038/ni.2117 21934668 PMC3541830

[B144] Kim Y. Sharov A. A. McDole K. Cheng M. Hao H. Fan C.-M. (2011). Mouse B-type lamins are required for proper organogenesis but not by embryonic stem cells. Science 334, 1706–1710. 10.1126/science.1211222 22116031 PMC3306219

[B145] Kim Y. Zheng X. Zheng Y. (2013). Proliferation and differentiation of mouse embryonic stem cells lacking all lamins. Cell Res. 23, 1420–1423. 10.1038/cr.2013.118 23979018 PMC3847566

[B146] Kinoshita T. Nagamatsu G. Kosaka T. Takubo K. Hotta A. Ellis J.. (2011). Ataxia-telangiectasia mutated (ATM) deficiency decreases reprogramming efficiency and leads to genomic instability in iPS cells. Biochem. Biophys. Res. Commun. 407, 321–326. 10.1016/j.bbrc.2011.03.013 21385566

[B147] Kuga T. Nie H. Kazami T. Satoh M. Matsushita K. Nomura F.. (2014). Lamin B2 prevents chromosome instability by ensuring proper mitotic chromosome segregation. Oncogenesis 3:e94. 10.1038/oncsis.2014.6 24637494 PMC4038388

[B148] Kulashreshtha M. Mehta I. S. Kumar P. Rao B. J. (2016). Chromosome territory relocation during DNA repair requires nuclear myosin 1 recruitment to chromatin mediated by ⋎-H2AX signaling. Nucleic Acids Res. 44, 8272–8291. 10.1093/nar/gkw573 27365048 PMC5041470

[B149] Kuroda M. Tanabe H. Yoshida K. Oikawa K. Saito A. Kiyuna T.. (2004). Alteration of chromosome positioning during adipocyte differentiation. J. Cell Sci. 117, 5897–5903. 10.1242/jcs.01508 15537832

[B150] Laguri C. Gilquin B. Wolff N. Romi-Lebrun R. Courchay K. Callebaut I.. (2001). Structural characterization of the LEM motif common to three human inner nuclear membrane proteins. Structure 9, 503–511. 10.1016/S0969-2126(01)00611-6 11435115

[B151] Lamm N. Ben-David U. Golan-Lev T. Storchová Z. Benvenisty N. Kerem B. (2016). Genomic instability in human pluripotent stem cells arises from replicative stress and chromosome condensation defects cell stem cell genomic instability in human pluripotent stem cells arises from replicative stress and chromosome condensation defects. Cell Stem Cell 18, 253–261. 10.1016/j.stem.2015.11.003 26669899

[B152] Lee J.-H. Hart S. R. L. Skalnik D. G. (2004). Histone deacetylase activity is required for embryonic stem cell differentiation. Genesis 38, 32–38. 10.1002/gene.10250 14755802

[B153] Lee J.-H. Paull T. T. (2007). Activation and regulation of ATM kinase activity in response to DNA double-strand breaks. Oncogene 26, 7741–7748. 10.1038/sj.onc.1210872 18066086

[B154] Lehner C. F. Stick R. Eppenberger H. M. Nigg E. A. (1987). Differential expression of nuclear lamin proteins during chicken development. J. Cell Biol. 105, 577–587. 3301871 10.1083/jcb.105.1.577 PMC2114895

[B155] Li F. Ackermann E. J. Bennett C. F. Rothermel A. L. Plescia J. Tognin S.. (1999). Pleiotropic cell-division defects and apoptosis induced by interference with survivin function. Nat. Cell Biol. 1, 461–466. 10587640 10.1038/70242

[B156] Li T. Zhao H. Han X. Yao J. Zhang L. Guo Y.. (2017). The spontaneous differentiation and chromosome loss in iPSCs of human trisomy 18 syndrome. Cell Death Dis. 8, e3149. 10.1038/cddis.2017.565 29072700 PMC5680928

[B157] Lieberman-Aiden E. van Berkum N. L. Williams L. Imakaev M. Ragoczy T. Telling A.. (2009). Comprehensive mapping of long-range interactions reveals folding principles of the human genome. Science 326, 289–293. 10.1126/science.1181369 19815776 PMC2858594

[B158] Lin F. Worman H. J. (1995). Structural organization of the human gene (LMNB1) encoding nuclear lamin B1. Genomics 27, 230–236. 10.1006/geno.1995.1036 7557986

[B159] Lin T. Chao C. Saito S. Mazur S. J. Murphy M. E. Appella E.. (2005). p53 induces differentiation of mouse embryonic stem cells by suppressing Nanog expression. Nat. Cell Biol. 7, 165–171. 10.1038/ncb1211 15619621

[B160] Lin Y.C. Benner C. Mansson R. Heinz S. Miyazaki K. Miyazaki M.. (2012). Global changes in the nuclear positioning of genes and intra- and interdomain genomic interactions that orchestrate B cell fate. Nat. Immunol. 13, 1196–1204. 10.1038/ni.2432 23064439 PMC3501570

[B161] Lindahl T. (1993). Instability and decay of the primary structure of DNA. Nature 362, 709–715. 10.1038/362709a0 8469282

[B162] Lisaingo K. Uringa E.-J. Lansdorp P. M. (2014). Resolution of telomere associations by TRF1 cleavage in mouse embryonic stem cells. Mol. Biol. Cell 25, 1958–1968. 10.1091/mbc.E13-10-0564 24829382 PMC4072570

[B163] Liu D. O'Connor M. S. Qin J. Songyang Z. (2004). Telosome, a mammalian telomere-associated complex formed by multiple telomeric proteins. J. Biol. Chem. 279, 51338–51342. 10.1074/jbc.M409293200 15383534

[B164] Lorimore S. A. Kadhim M. A. Pocock D. A. Papworth D. Stevens D. L. Goodhead D. T.. (1998). Chromosomal instability in the descendants of unirradiated surviving cells after alpha-particle irradiation. Proc. Natl. Acad. Sci. U.S.A. 95, 5730–5733. 9576952 10.1073/pnas.95.10.5730 PMC20447

[B165] Lu J. Li H. Baccei A. Sasaki T. Gilbert D. M. Lerou P. H. (2016). Influence of ATM -mediated DNA damage response on genomic variation in human induced pluripotent stem cells. Stem Cells Dev. 25, 740–747. 10.1089/scd.2015.0393 26935587 PMC4854209

[B166] Lu X. Shaw C. A. Patel A. Li J. Cooper M. L. Wells W. R.. (2007). Clinical implementation of chromosomal microarray analysis: summary of 2513 postnatal cases. PLoS ONE 2:e327. 10.1371/journal.pone.0000327 17389918 PMC1828620

[B167] Luu P. L. Gerovska D. Schöler H. R. Araúzo-Bravo M. J. (2018). Rules governing the mechanism of epigenetic reprogramming memory. Epigenomics 10, 149–174. 10.2217/epi-2017-0098 29334780

[B168] Ma L. Tsai M.-Y. Wang S. Lu B. Chen R. Yates J. R. III. (2009). Requirement for Nudel and dynein for assembly of the lamin B spindle matrix. Nat. Cell Biol. 11, 247–256. 10.1038/ncb1832 19198602 PMC2699591

[B169] Mahy N. L. Perry P. E. Bickmore W. A. (2002). Gene density and transcription influence the localization of chromatin outside of chromosome territories detectable by FISH. J. Cell Biol. 159, 753–763. 10.1083/jcb.200207115 12473685 PMC2173389

[B170] Maitra A. Arking D. E. Shivapurkar N. Ikeda M. Stastny V. Kassauei K.. (2005). Genomic alterations in cultured human embryonic stem cells. Nat. Genet. 37, 1099–1103. 10.1038/ng1631 16142235

[B171] Mallon B. S. Hamilton R. S. Kozhich O. A. Johnson K. R. Fann Y. C. Rao M. S.. (2014). Comparison of the molecular profiles of human embryonic and induced pluripotent stem cells of isogenic origin. Stem Cell Res. 12, 376–386. 10.1016/j.scr.2013.11.010 24374290 PMC4157340

[B172] Malone C. J. Misner L. Le Bot N. Tsai M.-C. Campbell J. M. Ahringer J.. (2003). The *C. elegans* hook protein, ZYG-12, mediates the essential attachment between the centrosome and nucleus. Cell 115, 825–836. 10.1016/S0092-8674(03)00985-1 14697201

[B173] Manning M. Hudgins L. Professional Practice Guidelines Committee (2010). Array-based technology and recommendations for utilization in medical genetics practice for detection of chromosomal abnormalities. Genet. Med. 12, 742–745. 10.1097/GIM.0b013e3181f8baad 20962661 PMC3111046

[B174] Marión R. M. Strati K. Li H. Murga M. Blanco R. Ortega S.. (2009). A p53-mediated DNA damage response limits reprogramming to ensure iPS cell genomic integrity. Nature 460, 1149–1153. 10.1038/nature08287 19668189 PMC3624089

[B175] Mariotti L. G. Pirovano G. Savage K. I. Ghita M. Ottolenghi A. Prise K. M.. (2013). Use of the γ-H2AX assay to investigate DNA repair dynamics following multiple radiation exposures. PLoS ONE 8:e79541. 10.1371/journal.pone.0079541 24312182 PMC3843657

[B176] Mattout A. Biran A. Meshorer E. (2011). Global epigenetic changes during somatic cell reprogramming to iPS cells. J. Mol. Cell Biol. 3, 341–350. 10.1093/jmcb/mjr028 22044880

[B177] Mayshar Y. Ben-David U. Lavon N. Biancotti J.-C. Yakir B. Clark A. T.. (2010). Identification and classification of chromosomal aberrations in human induced pluripotent stem Cell Stem Cell 7, 521–531. 10.1016/j.stem.2010.07.017 20887957

[B178] Mehta I. S. Amira M. Harvey A. J. Bridger J. M. (2010). Rapid chromosome territory relocation by nuclear motor activity in response to serum removal in primary human fibroblasts. Genome Biol. 11:R5. 10.1186/gb-2010-11-1-r5 20070886 PMC2847717

[B179] Menten B. Maas N. Thienpont B. Buysse K. Vandesompele J. Melotte C.. (2006). Emerging patterns of cryptic chromosomal imbalance in patients with idiopathic mental retardation and multiple congenital anomalies: a new series of 140 patients and review of published reports. J. Med. Genet. 43, 625–633. 10.1136/jmg.2005.039453 16490798 PMC2564583

[B180] Merkle F. T. Ghosh S. Kamitaki N. Mitchell J. Avior Y. Mello C.. (2017). Human pluripotent stem cells recurrently acquire and expand dominant negative P53 mutations. Nature 545, 229–233. 10.1038/nature22312 28445466 PMC5427175

[B181] Mertzanidou A. Wilton L. Cheng J. Spits C. Vanneste E. Moreau Y.. (2013). Microarray analysis reveals abnormal chromosomal complements in over 70% of 14 normally developing human embryos. Hum. Reprod. 28, 256–264. 10.1093/humrep/des362 23054067

[B182] Meshorer E. Gruenbaum Y. (2008). Gone with the Wnt/Notch: stem cells in laminopathies, progeria, and aging. J. Cell Biol. 181, 9–13. 10.1083/jcb.200802155 18378774 PMC2287275

[B183] Meshorer E. Misteli T. (2006). Chromatin in pluripotent embryonic stem cells and differentiation. Nat. Rev. Mol. Cell. Biol. 7, 540–546. 10.1038/nrm1938 16723974

[B184] Meshorer E. Yellajoshula D. George E. Scambler P. J. Brown D. T. Misteli T. (2006). Hyperdynamic plasticity of chromatin proteins in pluripotent embryonic stem cells. Dev. Cell 10, 105–116. 10.1016/j.devcel.2005.10.017 16399082 PMC1868458

[B185] Miller D. T. Adam M. P. Aradhya S. Biesecker L. G. Brothman A. R. Carter N. P. (2010). Consensus statement: chromosomal microarray is a first-tier clinical diagnostic test for individuals with developmental disabilities or congenital anomalies. Am. J. Hum. Genet. 86, 749–764. 10.1016/j.ajhg.2010.04.006 20466091 PMC2869000

[B186] Minissi S. Gustavino B. Degrassi F. Tanzarella C. Rizzoni M. (1999). Effect of cytochalasin B on the induction of chromosome missegregation by colchicine at low concentrations in human lymphocytes. Mutagenesis 14, 43–49. 10474820 10.1093/mutage/14.1.43

[B187] Mitalipova M. M. Rao R. R. Hoyer D. M. Johnson J. A. Meisner L. F. Jones K. L.. (2005). Preserving the genetic integrity of human embryonic stem cells. Nat. Biotechnol. 23, 19–20. 10.1038/nbt0105-19 15637610

[B188] Miura M. Miura Y. Padilla-Nash H. M. Molinolo A. A. Fu B. Patel V.. (2006). Accumulated chromosomal instability in murine bone marrow mesenchymal stem cells leads to malignant transformation. Stem Cells 24, 1095–1103. 10.1634/stemcells.2005-0403 16282438

[B189] Momcilovic O. Knobloch L. Fornsaglio J. Varum S. Easley C. Schatten G. (2010). DNA damage responses in human induced pluripotent stem cells and embryonic stem cells. PLoS ONE 5:e13410. 10.1371/journal.pone.0013410 20976220 PMC2955528

[B190] Mull A. N. Klar A. Navara C. S. (2014). Differential localization and high expression of SURVIVIN splice variants in human embryonic stem cells but not in differentiated cells implicate a role for SURVIVIN in pluripotency. Stem Cell Res. 12, 539–549. 10.1016/j.scr.2014.01.002 24487129

[B191] Munné S. Alikani M. Tomkin G. Grifo J. Cohen J. (1995). Embryo morphology, developmental rates, and maternal age are correlated with chromosome abnormalities. Fertil. Steril. 64, 382–391. 7615118

[B192] Munné S. Magli C. Bahçe M. Fung J. Legator M. Morrison L.. (1998). Preimplantation diagnosis of the aneuploidies most commonly found in spontaneous abortions and live births: XY, 13, 14, 15, 16, 18, 21, 22. Prenat. Diagn. 18, 1459–1466. 9949446 10.1002/(sici)1097-0223(199812)18:13<1459::aid-pd514>3.0.co;2-v

[B193] Na J. Baker D. Zhang J. Andrews P. W. Barbaric I. (2014). Aneuploidy in pluripotent stem cells and implications for cancerous transformation. Protein Cell 5, 569–579. 10.1007/s13238-014-0073-9 24899134 PMC4130921

[B194] Nagaria P. K. Robert C. Park T. S. Huo J. S. Zambidis E. T. Rassool F. V. (2016). High-fidelity reprogrammed human IPSCs have a high efficacy of DNA repair and resemble hESCs in their MYC transcriptional signature. Stem Cells Int. 2016, 1–14. 10.1155/2016/3826249 27688775 PMC5023833

[B195] Nagasawa H. Little J. B. (1992). Induction of sister chromatid exchanges by extremely low doses of alpha-particles. Cancer Res. 52, 6394–6396. 1423287

[B196] Nakamura T. M. Cech T. R. (1998). Reversing time: origin of telomerase. Cell 92, 587–590. 10.1016/S0092-8674(00)81123-X 9506510

[B197] Nelson G. Wordsworth J. Wang C. Jurk D. Lawless C. Martin-Ruiz C.. (2012). A senescent cell bystander effect: senescence-induced senescence. Aging Cell 11, 345–349. 10.1111/j.1474-9726.2012.00795.x 22321662 PMC3488292

[B198] Nera B. Huang H.-S. Lai T. Xu L. (2015). Elevated levels of TRF2 induce telomeric ultrafine anaphase bridges and rapid telomere deletions. Nat. Commun. 6:10132. 10.1038/ncomms10132 26640040 PMC4686832

[B199] Noatynska A. Gotta M. Meraldi P. (2012). Mitotic spindle (DIS)orientation and DISease: cause or consequence? J. Cell Biol. 199, 1025–1035. 10.1083/jcb.201209015 23266953 PMC3529530

[B200] Nora E. P. Lajoie B. R. Schulz E. G. Giorgetti L. Okamoto I. Servant N.. (2012). Spatial partitioning of the regulatory landscape of the X-inactivation centre. Nature 485, 381–385. 10.1038/nature11049 22495304 PMC3555144

[B201] Norppa H. Falck G. C.-M. (2003). What do human micronuclei contain? Mutagenesis 18, 221–233. 10.1093/mutage/18.3.221 12714687

[B202] Novik V. Moulton E. B. Sisson M. E. Shrestha S. L. Tran K. D. Stern H. J.. (2014). The accuracy of chromosomal microarray testing for identification of embryonic mosaicism in human blastocysts. Mol. Cytogenet. 7:18. 10.1186/1755-8166-7-18 24581286 PMC3944884

[B203] Ogilvie C. M. Donaghue C. Fox S. P. Docherty Z. Mann K. (2005). Rapid prenatal diagnosis of aneuploidy using quantitative fluorescence-PCR (QF-PCR). J. Histochem. Cytochem. 53, 285–288. 10.1369/jhc.4B6409.2005 15750003

[B204] Okae H. Chiba H. Hiura H. Hamada H. Sato A. Utsunomiya T.. (2014). Genome-wide analysis of DNA methylation dynamics during early human development. PLoS Genet. 10:e1004868. 10.1371/journal.pgen.1004868 25501653 PMC4263407

[B205] Olson E. N. Nordheim A. (2010). Linking actin dynamics and gene transcription to drive cellular motile functions. Nat. Rev. Mol. Cell Biol. 11, 353–365. 10.1038/nrm2890 20414257 PMC3073350

[B206] O'Neill L. P. Turner B. M. (1995). Histone H4 acetylation distinguishes coding regions of the human genome from heterochromatin in a differentiation-dependent but transcription-independent manner. EMBO J. 14, 3946–3957. 7664735 10.1002/j.1460-2075.1995.tb00066.x PMC394473

[B207] Orsztynowicz M. Lechniak D. Pawlak P. Kociucka B. Kubickova S. Cernohorska H.. (2017). Changes in chromosome territory position within the nucleus reflect alternations in gene expression related to embryonic lineage specification. PLoS ONE 12:e0182398. 10.1371/journal.pone.0182398 28767705 PMC5540545

[B208] Pajerowski J. D. Dahl K. N. Zhong F. L. Sammak P. J. Discher D. E. (2007). Physical plasticity of the nucleus in stem cell differentiation. Proc. Natl. Acad. Sci. U.S.A. 104, 15619–15624. 10.1073/pnas.0702576104 17893336 PMC2000408

[B209] Palm W. de Lange T. (2008). How shelterin protects mammalian telomeres. Annu. Rev. Genet. 42, 301–334. 10.1146/annurev.genet.41.110306.130350 18680434

[B210] Papp B. Plath K. (2011). Reprogramming to pluripotency: stepwise resetting of the epigenetic landscape. Cell Res. 21, 486–501. 10.1038/cr.2011.28 21321600 PMC3193418

[B211] Parada L. A. McQueen P. G. Misteli T. (2004). Tissue-specific spatial organization of genomes. Genome Biol. 5:R44. 10.1186/gb-2004-5-7-r44 15239829 PMC463291

[B212] Parada L. A. Misteli T. (2002). Chromosome positioning in the interphase nucleus. Trends Cell Biol. 12, 425–432. 10.1016/S0962-8924(02)02351-6 12220863

[B213] Pardo B. Marcand S. (2005). Rap1 prevents telomere fusions by nonhomologous end joining. EMBO J. 24, 3117–3127. 10.1038/sj.emboj.7600778 16096640 PMC1201357

[B214] Pekovic V. Hutchison C. J. (2008). Adult stem cell maintenance and tissue regeneration in the ageing context: the role for A-type lamins as intrinsic modulators of ageing in adult stem cells and their niches. J. Anat. 213, 5–25. 10.1111/j.1469-7580.2008.00928.x 18638067 PMC2475560

[B215] Peric-Hupkes D. Meuleman W. Pagie L. Bruggeman S. W. M. Solovei I. Brugman W.. (2010). Molecular maps of the reorganization of genome-nuclear lamina interactions during differentiation. Mol. Cell 38, 603–613. 10.1016/j.molcel.2010.03.016 20513434 PMC5975946

[B216] Peric-Hupkes D. van Steensel B. (2010). Role of the nuclear lamina in genome organization and gene expression. Cold Spring Harb. Symp. Quant. Biol. 75, 517–524. 10.1101/sqb.2010.75.014 21209388

[B217] Perovanovic J. Dell'Orso S. Gnochi V. F. Jaiswal J. K. Sartorelli V. Vigouroux C.. (2016). Laminopathies disrupt epigenomic developmental programs and cell fate. Sci. Transl. Med. 8:335ra58. 10.1126/scitranslmed.aad4991 27099177 PMC4939618

[B218] Peterson S. E. Loring J. F. (2014). Genomic instability in pluripotent stem cells: implications for clinical applications. J. Biol. Chem. 289, 4578–4584. 10.1074/jbc.R113.516419 24362040 PMC3931019

[B219] Phillips-Cremins J. E. (2014). Unraveling architecture of the pluripotent genome. Curr. Opin. Cell Biol. 28, 96–104. 10.1016/j.ceb.2014.04.006 24813689

[B220] Pollard K. M. Chan E. K. Grant B. J. Sullivan K. F. Tan E. M. Glass C. A. (1990). *In vitro* posttranslational modification of lamin B cloned from a human T-cell line. Mol. Cell. Biol. 10, 2164–2175. 2325650 10.1128/mcb.10.5.2164-2175.1990 PMC360564

[B221] Prokocimer M. Davidovich M. Nissim-Rafinia M. Wiesel-Motiuk N. Bar D. Z. Barkan R.. (2009). Nuclear lamins: key regulators of nuclear structure and activities. J. Cell. Mol. Med. 13, 1059–1085. 10.1111/j.1582-4934.2008.00676.x 19210577 PMC4496104

[B222] Pucci F. Gardano L. Harrington L. (2013). Short telomeres in ESCs lead to unstable differentiation. Cell Stem Cell 12, 479–486. 10.1016/j.stem.2013.01.018 23561444 PMC3629568

[B223] Quyn A. J. Appleton P. L. Carey F. A. Steele R. J. C. Barker N. Clevers H.. (2010). Spindle orientation bias in gut epithelial stem cell compartments is lost in precancerous tissue. Cell Stem Cell 6, 175–181. 10.1016/j.stem.2009.12.007 20144789

[B224] Ranade D. Koul S. Thompson J. Prasad K. B. Sengupta K. (2017). Chromosomal aneuploidies induced upon Lamin B2 depletion are mislocalized in the interphase nucleus. Chromosoma 126, 223–244. 10.1007/s00412-016-0580-y 26921073 PMC5371638

[B225] Razafsky D. Hodzic D. (2009). Bringing KASH under the SUN: the many faces of nucleo-cytoskeletal connections. J. Cell Biol. 186, 461–472. 10.1083/jcb.200906068 19687252 PMC2733748

[B226] Reuter V. E. (2005). Origins and molecular biology of testicular germ cell tumors. Mod. Pathol. 18 (Suppl. 2), S51–S60. 10.1038/modpathol.3800309 15761466

[B227] Rober R. A. Weber K. Osborn M. (1989). Differential timing of nuclear lamin A/C expression in the various organs of the mouse embryo and the young animal: a developmental study. Development 105, 365–378. 2680424 10.1242/dev.105.2.365

[B228] Robson M. I. I. de Las Heras J. I. Czapiewski R. Lê Thành P. Booth D. G. G. Kelly D. A. A.. (2016). Tissue-specific gene repositioning by muscle nuclear membrane proteins enhances repression of critical developmental genes during myogenesis. Mol. Cell 62, 834–847. 10.1016/j.molcel.2016.04.035 27264872 PMC4914829

[B229] Rohrabaugh S. Mantel C. Broxmeyer H. E. (2008). Mouse hematopoietic stem cells, unlike human and mouse embryonic stem cells, exhibit checkpoint-apoptosis coupling. Stem Cells Dev. 17, 1017–1020. 10.1089/scd.2007.0260 18788999 PMC2818989

[B230] Rönn R. E. Guibentif C. Saxena S. Woods N.-B. (2017). Reactive oxygen species impair the function of CD90 ^+^ hematopoietic progenitors generated from human pluripotent stem cells. Stem Cells 35, 197–206. 10.1002/stem.2503 27641910

[B231] Sachdev N. M. Maxwell S. M. Besser A. G. Grifo J. A. (2017). Diagnosis and clinical management of embryonic mosaicism. Fertil. Steril. 107, 6–11. 10.1016/j.fertnstert.2016.10.006 27842993

[B232] Sachdeva K. Discutido R. Albuz F. Almekosh R. Peramo B. (2017). Validation of next-generation sequencer for 24-chromosome aneuploidy screening in human embryos. Genet. Test. Mol. Biomarkers 21, 674–680. 10.1089/gtmb.2017.0108 28885040

[B233] Salníková M. Kozubek S. Lukášová E. Bártová E. Jirsová P. Cafourková A. (2000). Spatial arrangement of genes, centromeres and chromosomes in human blood cell nuclei and its changes during the cell cycle, differentiation and after irradiation. Chromosom. Res. 8, 487–499. 10.1023/A:1009267605580 11032319

[B234] Salsman J. Rapkin L. M. Margam N. N. Duncan R. Bazett-Jones D. P. Dellaire G. (2017). Myogenic differentiation triggers PML nuclear body loss and DAXX relocalization to chromocentres. Cell Death Dis. 8, e2724. 10.1038/cddis.2017.151 28358373 PMC5386546

[B235] Sartore R. C. Campos P. B. Trujillo C. A. Ramalho B. L. Negraes P. D. Paulsen B. S.. (2011). Retinoic acid-treated pluripotent stem cells undergoing neurogenesis present increased aneuploidy and micronuclei formation. PLoS ONE 6:e20667. 10.1371/journal.pone.0020667 21674001 PMC3108948

[B236] Sawant S. G. Randers-Pehrson G. Geard C. R. Brenner D. J. Hall E. J. (2001). The bystander effect in radiation oncogenesis: I. Transformation in C3H 10T1/2 cells *in vitro* can be initiated in the unirradiated neighbors of irradiated cells. Radiat. Res. 155, 397–401. 10.1667/0033-7587(2001)155[0397:TBEIRO]2.0.CO;2 11182789

[B237] Scaffidi P. Misteli T. (2008). Lamin A-dependent misregulation of adult stem cells associated with accelerated ageing. Nat. Cell Biol. 10, 452–459. 10.1038/ncb1708 18311132 PMC2396576

[B238] Schneider R. P. Garrobo I. Foronda M. Palacios J. A. Marión R. M. Flores I.. (2013). TRF1 is a stem cell marker and is essential for the generation of induced pluripotent stem cells. Nat. Commun. 4:1946. 10.1038/ncomms2946 23735977

[B239] Sellner L. N. Taylor G. R. (2004). MLPA and MAPH: new techniques for detection of gene deletions. Hum. Mutat. 23, 413–419. 10.1002/humu.20035 15108271

[B240] Sexton T. Yaffe E. Kenigsberg E. Bantignies F. Leblanc B. Hoichman M.. (2012). Three-dimensional folding and functional organization principles of the Drosophila genome. Cell 148, 458–472. 10.1016/j.cell.2012.01.010 22265598

[B241] Sfeir A. Kosiyatrakul S. T. Hockemeyer D. MacRae S. L. Karlseder J. Schildkraut C. L.. (2009). Mammalian telomeres resemble fragile sites and require TRF1 for efficient replication. Cell 138, 90–103. 10.1016/j.cell.2009.06.021 19596237 PMC2723738

[B242] Shaffer L. G. (2007). Molecular cytogenetic and rapid aneuploidy detection methods in prenatal diagnosis. Am. J. Med. Genet. Part C Semin. Med. Genet. 145, 87–98. 10.1002/ajmg.c.30114 17290441

[B243] Simon D. N. Wilson K. L. (2011). The nucleoskeleton as a genome-associated dynamic network of networks. Nat. Rev. Mol. Cell Biol. 12, 695–708. 10.1038/nrm3207 21971041

[B244] Simon D. N. Zastrow M. S. Wilson K. L. (2010). Direct actin binding to A- and B-type lamin tails and actin filament bundling by the lamin A tail. Nucleus 1, 264–272. 10.4161/nucl.1.3.11799 21327074 PMC3027033

[B245] Singer Z. S. Yong J. Tischler J. Hackett J. A. Altinok A. Surani M. A. (2014). Dynamic heterogeneity and DNA methylation in embryonic stem cells. Mol. Cell 55, 319–331. 10.1016/j.molcel.2014.06.029 25038413 PMC4104113

[B246] Smith E. R. Meng Y. Moore R. Tse J. D. Xu A. G. Xu X.-X. (2017). Nuclear envelope structural proteins facilitate nuclear shape changes accompanying embryonic differentiation and fidelity of gene expression. BMC Cell Biol. 18:8. 10.1186/s12860-017-0125-0 28088180 PMC5237523

[B247] Solomon S. Pitossi F. Rao M. S. (2015). Banking on iPSC–is it doable and is it worthwhile. Stem Cell Rev. 11, 1–10. 10.1007/s12015-014-9574-4 25516409 PMC4333229

[B248] Solovei I. Wang A. S. Thanisch K. Schmidt C. S. Krebs S. Zwerger M.. (2013). LBR and Lamin A/C sequentially tether peripheral heterochromatin and inversely regulate differentiation. Cell 152, 584–598. 10.1016/j.cell.2013.01.009 23374351

[B249] Song W. K. Park K.-M. Kim H.-J. Lee J. H. Choi J. Chong S. Y.. (2015). Treatment of macular degeneration using embryonic stem cell-derived retinal pigment epithelium: preliminary results in Asian patients. Stem Cell Rep. 4, 860–872. 10.1016/j.stemcr.2015.04.005 25937371 PMC4437471

[B250] Spann T. P. Goldman A. E. Wang C. Huang S. Goldman R. D. (2002). Alteration of nuclear lamin organization inhibits RNA polymerase II–dependent transcription. J. Cell Biol. 156, 603–608. 10.1083/jcb.200112047 11854306 PMC2174089

[B251] Sperger J. M. Chen X. Draper J. S. Antosiewicz J. E. Chon C. H. Jones S. B.. (2003). Gene expression patterns in human embryonic stem cells and human pluripotent germ cell tumors. Proc. Natl. Acad. Sci. U.S.A. 100, 13350–13355. 10.1073/pnas.2235735100 14595015 PMC263817

[B252] Sperka T. Wang J. Rudolph K. L. (2012). DNA damage checkpoints in stem cells, ageing and cancer. Nat. Rev. Mol. Cell Biol. 13, 579–590. 10.1038/nrm3420 22914294

[B253] Stewart C. Burke B. (1987). Teratocarcinoma stem cells and early mouse embryos contain only a single major lamin polypeptide closely resembling lamin B. Cell 51, 383–392. 10.1016/0092-8674(87)90634-9 3311384

[B254] Stopper H. Schmitt E. Gregor C. Mueller S. O. Fischer W. H. (2003). Increased cell proliferation is associated with genomic instability: elevated micronuclei frequencies in estradiol-treated human ovarian cancer cells. Mutagenesis 18, 243–247. 10.1093/mutage/18.3.243 12714689

[B255] Suh Y.-A. Arnold R. S. Lassegue B. Shi J. Xu X. Sorescu D.. (1999). Cell transformation by the superoxide-generating oxidase Mox1. Nature 401, 79–82. 10.1038/43459 10485709

[B256] Summersgill B. M. Jafer O. Wang R. Goker H. Niculescu-Duvaz I. Huddart R.. (2001). Definition of chromosome aberrations in testicular germ cell tumor cell lines by 24-color karyotyping and complementary molecular cytogenetic analyses. Cancer Genet. Cytogenet. 128, 120–129. 10.1016/S0165-4608(01)00414-9 11463450

[B257] Suvorova I. I. Grigorash B. B. Chuykin I. A. Pospelova T. V. Pospelov V. A. (2016). G1 checkpoint is compromised in mouse ESCs due to functional uncoupling of p53-p21Waf1 signaling. Cell Cycle 15, 52–63. 10.1080/15384101.2015.1120927 26636245 PMC4825740

[B258] Swift J. Discher D. E. (2014). The nuclear lamina is mechano-responsive to ECM elasticity in mature tissue. J. Cell Sci. 127, 3005–3015. 10.1242/jcs.149203 24963133 PMC4095853

[B259] Takagi Y. Takahashi J. Saiki H. Morizane A. Hayashi T. Kishi Y.. (2005). Dopaminergic neurons generated from monkey embryonic stem cells function in a Parkinson primate model. J. Clin. Invest. 115, 102–109. 10.1172/JCI21137 15630449 PMC539189

[B260] Takahashi K. Tanabe K. Ohnuki M. Narita M. Ichisaka T. Tomoda K.. (2007). Induction of pluripotent stem cells from adult human fibroblasts by defined factors. Cell 131, 861–872. 10.1016/J.CELL.2007.11.019 18035408

[B261] Takahashi K. Yamanaka S. (2006). Induction of pluripotent stem cells from mouse embryonic and adult fibroblast cultures by defined factors. Cell 126, 663–676. 10.1016/j.cell.2006.07.024 16904174

[B262] Tanabe H. Müller S. Neusser M. von Hase J. Calcagno E. Cremer M.. (2002). Evolutionary conservation of chromosome territory arrangements in cell nuclei from higher primates. Proc. Natl. Acad. Sci. U.S.A. 99, 4424–4429. 10.1073/pnas.072618599 11930003 PMC123664

[B263] Tanaka T. Shimizu N. (2000). Induced detachment of acentric chromatin from mitotic chromosomes leads to their cytoplasmic localization at G(1) and the micronucleation by lamin reorganization at S phase. J. Cell Sci. 113 (Pt 4), 697–707. 10652262 10.1242/jcs.113.4.697

[B264] Taylor C. J. Peacock S. Chaudhry A. N. Bradley J. A. Bolton E. M. (2012). Generating an iPSC Bank for HLA-matched tissue transplantation based on known donor and recipient HLA types. Cell Stem Cell 11, 147–152. 10.1016/j.stem.2012.07.014 22862941

[B265] Taylor T. H. Gitlin S. A. Patrick J. L. Crain J. L. Wilson J. M. Griffin D. K. (2014). The origin, mechanisms, incidence and clinical consequences of chromosomal mosaicism in humans. Hum. Reprod. Update 20, 571–581. 10.1093/humupd/dmu016 24667481

[B266] Theunissen T. W. Jaenisch R. (2017). Mechanisms of gene regulation in human embryos and pluripotent stem cells. Development 144, 4496–4509. 10.1242/dev.157404 29254992 PMC5769625

[B267] Tokunaga K. Saitoh N. Goldberg I. G. Sakamoto C. Yasuda Y. Yoshida Y.. (2014). Computational image analysis of colony and nuclear morphology to evaluate human induced pluripotent stem cells. Sci. Rep. 4:6996. 10.1038/srep06996 25385348 PMC4227026

[B268] Tsai M.-Y. Wang S. Heidinger J. M. Shumaker D. K. Adam S. A. Goldman R. D.. (2006). A mitotic Lamin B matrix induced by RanGTP required for spindle assembly. Science 311, 1887–1893. 10.1126/science.1122771 16543417

[B269] Turner M. Leslie S. Martin N. G. Peschanski M. Rao M. Taylor C. J.. (2013). Toward the development of a global induced pluripotent stem cell library. Cell Stem Cell 13, 382–384. 10.1016/j.stem.2013.08.003 24094319

[B270] Tusell L. Pampalona J. Soler D. Frías C. Genescà A. (2010). Different outcomes of telomere-dependent anaphase bridges. Biochem. Soc. Trans. 38, 1698–1703. 10.1042/BST0381698 21118150

[B271] Uchiyama Y. Nakashima M. Watanabe S. Miyajima M. Taguri M. Miyatake S.. (2016). Ultra–sensitive droplet digital PCR for detecting a low–prevalence somatic GNAQ mutation in Sturge–Weber syndrome. Sci. Rep. 6:22985. 10.1038/srep22985 26957145 PMC4783707

[B272] Uemura T. Ohkura H. Adachi Y. Morino K. Shiozaki K. Yanagida M. (1987). DNA topoisomerase II is required for condensation and separation of mitotic chromosomes in S. pombe. Cell 50, 917–925. 3040264 10.1016/0092-8674(87)90518-6

[B273] Utani K. Kawamoto J. Shimizu N. (2007). Micronuclei bearing acentric extrachromosomal chromatin are transcriptionally competent and may perturb the cancer cell phenotype. Mol. Cancer Res. 5, 695–704. 10.1158/1541-7786.MCR-07-0031 17606478

[B274] Vallabhaneni H. Lynch P. J. Chen G. Park K. Liu Y. Goehe R.. (2018). High basal levels of γH2AX in human induced pluripotent stem cells are linked to replication-associated DNA damage and repair. Stem Cells 36, 1501–1513. 10.1002/stem.2861 29873142 PMC6662168

[B275] van Echten-Arends J. Mastenbroek S. Sikkema-Raddatz B. Korevaar J. C. Heineman M. J. van der Veen F.. (2011). Chromosomal mosaicism in human preimplantation embryos: a systematic review. Hum. Reprod. Update 17, 620–627. 10.1093/humupd/dmr014 21531753

[B276] van Steensel B. Belmont A. S. (2017). Lamina-associated domains: links with chromosome architecture, heterochromatin, and gene repression. Cell 169, 780–791. 10.1016/j.cell.2017.04.022 28525751 PMC5532494

[B277] van Veghel-Plandsoen M. M. Wouters C. H. Kromosoeto J. N. R. den Ridder-Klünnen M. C. Halley D. J. J. van den Ouweland A. M. W. (2011). Multiplex ligation-depending probe amplification is not suitable for detection of low-grade mosaicism. Eur. J. Hum. Genet. 19, 1009–1012. 10.1038/ejhg.2011.60 21487440 PMC3179360

[B278] Volpi E. V. Chevret E. Jones T. Vatcheva R. Williamson J. Beck S.. (2000). Large-scale chromatin organization of the major histocompatibility complex and other regions of human chromosome 6 and its response to interferon in interphase nuclei. J. Cell Sci. 113 (Pt 9), 1565–1576. 10751148 10.1242/jcs.113.9.1565

[B279] Wang X. Allen T. D. May R. J. Lightfoot S. Houchen C. W. Huycke M. M. (2008). *Enterococcus faecalis* induces aneuploidy and tetraploidy in colonic epithelial cells through a bystander effect. Cancer Res. 68, 9909–9917. 10.1158/0008-5472.CAN-08-1551 19047172 PMC2596646

[B280] Wei Z. Gao F. Kim S. Yang H. Lyu J. An W.. (2013). Klf4 Organizes long-range chromosomal interactions with the Oct4 locus in reprogramming and pluripotency. Cell Stem Cell 13, 36–47. 10.1016/J.STEM.2013.05.010 23747203

[B281] Weierich C. Brero A. Stein S. von Hase J. Cremer C. Cremer T.. (2003). Three-dimensional arrangements of centromeres and telomeres in nuclei of human and murine lymphocytes. Chromosom. Res. 11, 485–502. 10.1023/A:1025016828544 12971724

[B282] Weimer R. Haaf T. Kriiger J. Poot M. Schmid M. (1992). Characterization of centromere arrangements and test for random distribution in Go, G1, S, G2, G1, and early S' phase in human lymphocytes. Hum. Genet. 88, 673–682. 1551672 10.1007/BF02265296

[B283] Weissbein U. Benvenisty N. Ben-David U. (2014). Genome maintenance in pluripotent stem cells. J. Cell Biol. 204, 153–163. 10.1083/jcb.201310135 24446481 PMC3897183

[B284] Weissbein U. Schachter M. Egli D. Benvenisty N. (2016). Analysis of chromosomal aberrations and recombination by allelic bias in RNA-Seq. Nat. Commun. 7, 12144. 10.1038/ncomms12144 27385103 PMC4941052

[B285] Wiblin A. E. Cui W. Clark A. J. Bickmore W. A. (2005). Distinctive nuclear organisation of centromeres and regions involved in pluripotency in human embryonic stem cells. J. Cell Sci. 118, 3861–3868. 10.1242/jcs.02500 16105879

[B286] WiCell (2017). The Combined Power of Karyotyping and aCGH. Available online at: https://www.wicell.org/media.acux/edb641e5-cb23-400a-9dcc-8f98e7e6bf07 (Accessed December 17, 2018).

[B287] Wolff N. Gilquin B. Courchay K. Callebaut I. Worman H. J. Zinn-Justin S. (2001). Structural analysis of emerin, an inner nuclear membrane protein mutated in X-linked Emery-Dreifuss muscular dystrophy. FEBS Lett. 501, 171–176. 10.1016/S0014-5793(01)02649-7 11470279

[B288] Woodbine L. Brunton H. Goodarzi A. A. Shibata A. Jeggo P. A. (2011). Endogenously induced DNA double strand breaks arise in heterochromatic DNA regions and require ataxia telangiectasia mutated and Artemis for their repair. Nucleic Acids Res. 39, 6986–6997. 10.1093/nar/gkr331 21596788 PMC3167608

[B289] Worman H. J. Bonne G. (2007). Laminopathies: a wide spectrum of human diseases Howard. Exp. Cell Res. 313, 2121–2133. 10.1016/j.yexcr.2007.03.028 17467691 PMC2964355

[B290] Wydner K. L. McNeil J. A. Lin F. Worman H. J. Lawrence J. B. (1996). Chromosomal assignment of human nuclear envelope protein genes LMNA, LMNB1, and LBR by fluorescence *in situ* hybridization. Genomics 32, 474–478. 10.1006/geno.1996.0146 8838815

[B291] Xiang B. Li A. Valentin D. Nowak N. J. Zhao H. Li P. (2008). Analytical and clinical validity of whole-genome oligonucleotide array comparative genomic hybridization for pediatric patients with mental retardation and developmental delay. Am. J. Med. Genet. Part A 146A, 1942–1954. 10.1002/ajmg.a.32411 18627053

[B292] Yamamori T. Yasui H. Yamazumi M. Wada Y. Nakamura Y. Nakamura H.. (2012). Ionizing radiation induces mitochondrial reactive oxygen species production accompanied by upregulation of mitochondrial electron transport chain function and mitochondrial content under control of the cell cycle checkpoint. Free Radic. Biol. Med. 53, 260–270. 10.1016/j.freeradbiomed.2012.04.033 22580337

[B293] Yan J.-B. Xu M. Xiong C. Zhou D.-W. Ren Z.-R. Huang Y.. (2011). Rapid screening for chromosomal aneuploidies using array-MLPA. BMC Med. Genet. 12:68. 10.1186/1471-2350-12-68 21575262 PMC3111339

[B294] Yang C. Przyborski S. Cooke M. J. Zhang X. Stewart R. Anyfantis G.. (2008). A key role for telomerase reverse transcriptase unit in modulating human embryonic stem cell proliferation, cell cycle dynamics, and *in vitro* differentiation. Stem Cells 26, 850–863. 10.1634/stemcells.2007-0677 18203676

[B295] Yang S. Lin G. Tan Y.-Q. Zhou D. Deng L.-Y. Cheng D.-H.. (2008). Tumor progression of culture-adapted human embryonic stem cells during long-term culture. Genes Chromosom. Cancer 47, 665–679. 10.1002/gcc.20574 18470900

[B296] Yao G. Xu J. Xin Z. Niu W. Shi S. Jin H.. (2016). Developmental potential of clinically discarded human embryos and associated chromosomal analysis. Sci. Rep. 6:23995. 10.1038/srep23995 27045374 PMC4820740

[B297] Yermilov V. Yoshie Y. Rubio J. Ohshima H. (1996). Effects of carbon dioxide/bicarbonate on induction of DNA single-strand breaks and formation of 8-nitroguanine, 8-oxoguanine and base-propenal mediated by peroxynitrite. FEBS Lett. 399, 67–70. 8980121 10.1016/s0014-5793(96)01288-4

[B298] Yoshihara M. Araki R. Kasama Y. Kawaji H. Hayashizaki Y. Correspondence Y. M.. (2017). Hotspots of *de novo* point mutations in induced pluripotent stem cells. Cell Rep. 21, 308–315. 10.1016/j.celrep.2017.09.060 29020618

[B299] Zastrow M. S. Vlcek S. Wilson K. L. (2004). Proteins that bind A-type lamins: integrating isolated clues. J. Cell Sci. 117, 979–987. 10.1242/jcs.01102 14996929

[B300] Zhang M. Cheng L. Jia Y. Liu G. Li C. Song S.. (2016). Aneuploid embryonic stem cells exhibit impaired differentiation and increased neoplastic potential. EMBO J. 35, 2285–2300. 10.15252/embj.201593103 27558554 PMC5090214

[B301] Zhang M. Wang L. An K. Cai J. Li G. Yang C.. (2018). Lower genomic stability of induced pluripotent stem cells reflects increased non-homologous end joining. Cancer Commun. 38, 49. 10.1186/s40880-018-0313-0 30045759 PMC6060453

[B302] Zhang R. Hao L. Wang L. Chen M. Li W. Li R.. (2013). Gene expression analysis of induced pluripotent stem cells from aneuploid chromosomal syndromes. BMC Genomics 14(Suppl. 5):S8. 10.1186/1471-2164-14-S5-S8 24564826 PMC3852284

[B303] Zhao R. Deibler R. W. Lerou P. H. Ballabeni A. Heffner G. C. Cahan P.. (2014). A nontranscriptional role for Oct4 in the regulation of mitotic entry. Proc. Natl. Acad. Sci. U.S.A. 111, 15768–15773. 10.1073/pnas.1417518111 25324523 PMC4226071

[B304] Zheng X. Kim Y. Zheng Y. (2015). Identification of lamin B-regulated chromatin regions based on chromatin landscapes. Mol. Biol. Cell 26, 2685–2697. 10.1091/mbc.E15-04-0210 25995381 PMC4501365

[B305] Zhou G. Meng S. Li Y. Ghebre Y. T. Cooke J. P. (2016). Optimal ROS signaling is critical for nuclear reprogramming. Cell Rep. 15, 919–925. 10.1016/j.celrep.2016.03.084 27117405 PMC4856580

[B306] Zuo B. Yang J. Wang F. Wang L. Yin Y. Dan J.. (2012). Influences of lamin A levels on induction of pluripotent stem cells. Biol. Open 1, 1118–1127. 10.1242/bio.20121586 23213392 PMC3507184

